# Review of 3D-Printed Cementitious and Geopolymer Permanent Formwork for Concrete Structures

**DOI:** 10.3390/ma19194031

**Published:** 2026-09-22

**Authors:** Muhammad Shaharyar Qaisar, Hocine Siad, Mohamed Lachemi, Obaid Mahmood, Mustafa Sahmaran

**Affiliations:** 1 Department of Civil Engineering, Toronto Metropolitan University, Toronto, ON M5B 2K3, Canada; mqaisar@torontomu.ca (M.S.Q.); mlachemi@torontomu.ca (M.L.); omahmoodi@torontomu.ca (O.M.); 2 Department of Civil Engineering, Hacettepe University, 06800 Ankara, Turkey; sahmaran@hacettepe.edu.tr

**Keywords:** three-dimensional concrete printing (3DCP), permanent formwork, printed-cast concrete, interfacial bonding, structural performance, durability, sustainability

## Abstract

**Highlights:**

**What are the main findings?**

- 3D-printed permanent formwork enables efficient printed-cast composite construction.
- Rheology and buildability govern printing stability and dimensional accuracy.
- Printed-cast interface quality is critical to composite structural performance.
- Printed formwork can enhance strength, ductility, and confinement of structural members.
- Geopolymer systems represent an emerging low-carbon option, but direct permanent formwork evidence remains very limited.

**What are the implications of the main findings?**

- Permanent formwork can reduce formwork use, labor demand, and construction waste.
- Interface engineering is essential for reliable composite structural action.
- Mix design must balance printability, mechanical performance, and durability.
- Standardized design and testing methods are needed for structural implementation.
- Long-term durability and full-scale validation remain key research priorities.

**Abstract:**

Three-dimensional concrete printing (3DCP) has emerged as a promising technology for developing stay-in-place cementitious and geopolymer permanent formwork systems. This review critically synthesizes current knowledge on printable materials, printability requirements, structural performance, interfacial bonding, durability, sustainability, and microstructural characteristics. Existing studies demonstrate that 3D-printed permanent formworks can contribute structurally by enhancing the axial, eccentric, and flexural performance of composite members through confinement, composite action, and effective load transfer between the printed shell and cast concrete core. Interfacial bonding is identified as a key factor governing structural performance, with layer height, interface geometry, surface roughness, and casting interval significantly affecting tensile and shear bond strengths. Microstructural studies further highlight the roles of pore characteristics, hydration development, and mechanical interlocking in interface formation. Although cementitious systems dominate current research, geopolymer permanent formworks remain comparatively underexplored despite their sustainability potential. Moreover, durability studies, life-cycle assessments, and direct comparisons between cementitious and geopolymer systems remain scarce. This review identifies key research gaps and outlines future directions toward performance-based design methodologies and the broader structural implementation of 3D-printed permanent formwork systems.

## 1. Introduction

Formwork is a temporary mold or support used to construct concrete elements [1]. It provides the required shape and support to freshly placed concrete until the concrete develops sufficient strength to support itself. Traditional formwork is typically made of rigid materials, such as timber and metal, and is commonly used to construct concrete structures with regular geometries [2]. However, although traditional formwork plays a crucial role in construction, it contributes significantly to overall material and labor costs, accounting for approximately 35–60% of the total construction project cost [3]. Furthermore, the majority of traditional formwork solutions are built around uniform, factory-produced, reusable flat panel modules, inherently limiting the complexity and diversity of shapes achievable in concrete construction [4,5,6,7,8,9]. Environmental impact is another limitation of current formwork systems, as the use of conventional timber results in significant waste generation and an increased environmental burden [10].

This situation has driven the need for innovative construction techniques that reduce material waste, lower labor costs, and improve overall geometric flexibility and sustainability [11,12,13]. One promising approach is three-dimensional concrete printing (3DCP), which can be used to produce permanent stay-in-place formwork. Three-dimensional concrete printing is an additive manufacturing (AM) technique that has recently gained traction in the construction industry due to its potential to automate construction and enable geometrically complex structures. The 3DCP formwork systems (3DCPFW) offer a cost-effective and sustainable alternative to conventional formwork, enabling the fabrication of complex geometries with minimal material use and near-zero waste. Extrusion-based concrete printing is the most commonly used technique in 3DCP and is currently the most established and widely used method for producing stay-in-place printed concrete formwork. This layer-by-layer deposition process in 3DCP enables the production of medium- to large-scale civil engineering elements and has been successfully used for structures such as bridges, building components, and housing units [14,15]. Although other cementitious additive manufacturing approaches, such as powder-based methods [16,17], have been explored within digital fabrication, their application to structurally relevant stay-in-place printed frames remains limited compared with extrusion-based 3DCP. Broader reviews also identify raw material selection and concrete type as central to the sustainability and performance of 3D concrete printing [4,18,19].

Unlike conventional casting, extrusion-based 3D concrete printing requires a careful balance between sufficient flowability during pumping and extrusion and rapid structural build-up after deposition. This requirement becomes more critical for 3DCP stay-in-place frames, where enhanced thixotropic behavior is needed to ensure shape stability and structural integrity during and after printing. Key rheological parameters, such as yield stress, viscosity, and thixotropy, must be carefully controlled, as they govern essential performance aspects, including pumpability, extrudability, shape retention, and interlayer bonding of the printed formwork [20,21]. Therefore, comprehensive rheological characterization, combined with performance-based evaluation under realistic processing conditions, is essential for optimizing 3DCP stay-in-place formwork systems, ensuring material consistency, and enabling reliable and scalable implementation in advanced construction applications. Previous studies on 3D-printed formwork have explored diverse fabrication techniques and material systems, including concrete-, clay-, polymer-, wax-, foam-, and sand-based materials produced through binder jetting [4]. These technologies have been implemented in columns, beams, slabs, and geometrically complex structural elements, offering greater design flexibility, efficient material utilization, and opportunities for hybrid construction. According to Jipa and Dillenburger [4], concrete- and foam-extrusion methods are particularly suitable for vertically extended elements, such as walls and columns, because of their higher structural stability. Comparative information among 3D-printed formwork systems remains limited; however, concrete- and clay-based systems have been reported to exhibit relatively low embodied energy compared with several other material systems [4]. In addition, 3D-printed concrete stay-in-place formwork can function not only as a casting mold for the infilled concrete but also as a durable protective layer that may enhance the overall durability of the resulting concrete structure [22].

Despite its recognized sustainability, economic, and practical advantages, research on 3DCP permanent (stay-in-place) formwork remains at an early stage of development. To the authors’ knowledge, no prior review has specifically focused on 3D concrete printing permanent formwork systems as its central theme. Existing reviews primarily address 3D-printed formwork in general, structural applications of 3DCP, or broader challenges related to its implementation, with permanent formwork systems only briefly discussed as an emerging application. For instance, the review by Jipa and Dillenburger [4] provides an overview of 3D-printed formwork across various materials and fabrication techniques, including permanent formwork concepts. However, a dedicated review specifically targeting 3DCP permanent formworks, considering their fresh properties requirements, printability constraints, interaction with infill concrete, structural performance, and durability as stay-in-place load-bearing systems, remains lacking. Therefore, a comprehensive and up-to-date review is needed to consolidate recent advancements, establish robust relationships between mixture design and fresh-driven printability, and assess the practical feasibility and in-place performance of 3DCP permanent formwork systems, ultimately supporting their technically reliable and scalable implementation in sustainable construction.

This review focuses on the recent advancements in 3D-printed cementitious and geopolymer formworks as stay-in-place, permanent systems for casting conventional concrete. The report examines the fundamental fresh properties, printing technologies, material compositions, and interfacial bonding between printed and cast concrete. It also examines durability performance, sustainability considerations, and existing research gaps to provide insights into the potential of 3D-printed formwork as a sustainable and multifunctional alternative to conventional systems. It is important to mention that, in this review, the term “3D-printed permanent formwork” is adopted as the principal terminology for the overall stay-in-place formwork system, whereas “printed shell” specifically refers to the 3D-printed component that remains integrated with the post-cast concrete. In addition, alkali-activated materials are considered the broader class of binder systems produced through alkali activation of suitable precursors, whereas the term “geopolymer” is retained for materials specifically identified as such in the original studies. Accordingly, the two terms are not used interchangeably.

## 2. Methodology of the Current Review

Scopus was selected as the bibliographic database for this review to provide a consistent source of indexed bibliographic records and author-keyword data for the literature screening and subsequent bibliometric analysis using VOSviewer (version 1.6.20). The Scopus search was last updated on 9 September 2026 and was applied to the article title, abstract, and keywords. The Boolean search combined terms related to 3D concrete printing, permanent formwork, and binder systems using the following search expression: “3D concrete printing” OR “3D-printed concrete” OR “additive manufacturing concrete” AND “stay-in-place” OR “permanent formwork” OR “printed shell” OR “printed mold” OR “hybrid casting” AND “cement” OR “geopolymer” OR “alkali-activated”. Both ‘geopolymer’ and ‘alkali-activated’ were included as search terms to capture the terminology used across the literature; however, these terms are not treated as synonymous in the technical discussion. The initial search retrieved 99 documents. Application of the publication-year range 2015–2026 reduced the dataset to 93 documents. The subject-area filter was subsequently restricted to Engineering and Materials Science, resulting in 86 documents. Restriction of the document type to articles and conference papers further reduced the dataset to 71 documents, and application of the English-language criterion resulted in a final bibliometric dataset of 69 documents. The resulting dataset of 69 publications was subsequently assessed for relevance to the technical scope of this review. Studies were considered eligible when they investigated 3D-printed cementitious or geopolymer permanent/stay-in-place formwork, printed shell-cast concrete systems, or related hybrid printed-cast structural systems, including their material composition, fresh-state and rheological behavior, printing parameters, mechanical and structural performance, interfacial bonding, durability, microstructure, or sustainability. Studies dealing exclusively with topics outside the scope of construction-scale 3D printing, permanent or hybrid formwork applications, conventional removable formwork, or non-cementitious additive manufacturing unrelated to construction formwork were considered for exclusion. Following the relevance assessment, all 69 publications satisfied the eligibility criteria and were retained for the technical synthesis. The 69 records were exported from Scopus in CSV format with complete bibliographic information and imported into VOSviewer for bibliometric analysis [23,24]. A co-occurrence analysis was performed using author keywords as the unit of analysis and the full-counting method. The minimum occurrence threshold was set to 2 occurrences per keyword, resulting in 41 keywords meeting the threshold and being included in the network visualization presented in Figure 1.

## 3. Background

### 3.1. 3D Printing Technology

To understand how 3DCP technology works, it is essential to comprehend the various printing methods, the rheological behavior of the printed material, and the criteria that affect printability. The concrete printing techniques can be classified into two main types: extrusion- and powder-based methods [25,26], although other less common approaches, such as shotcrete-based, binder jetting, and robotic assembly systems, have also been explored. In the extrusion-based method, pre-mixed materials are extruded layer-by-layer along a designated path [19]. This technique is commonly used for large-scale architectural structures with complex geometries. Within extrusion-based printing, two main processes are recognized: concrete printing and contour crafting, each with distinct applications [14]. Powder-based methods, on the other hand, utilize a liquid binder, which is deposited in layers over a powder (e.g., sand) to bind the powder particles into the desired geometry [25,26]. Common examples of powder-based printing techniques include the emerging objects and D-shape approaches [14]. Although this technique offers advantages, such as high printing resolution and great geometric freedom, its relatively slow printing speed makes it more suitable for off-site and small-scale manufacturing [14,25,26]. Figure 2 illustrates the key differences between extrusion- and powder-based printing methods.

### 3.2. Rheology of Printed Materials

Mastering 3D concrete printing requires a thorough understanding of the material’s rheological behavior. Rheology is the study of the deformation and flow of matter under applied forces [27], and it plays a vital role in determining the pumpability, extrudability, and buildability of 3D-printed concrete [28,29]. Different constitutive descriptions, including modified Bingham and parabolic models, have been evaluated for representing the nonlinear rheological response of fresh cementitious pastes [30]. Optimal pumpability is achieved by combining low plastic viscosity, which allows for smooth flow during pumping, with an adequate dynamic yield stress (the minimum shear stress required to maintain flow) to prevent segregation [29,31]. Furthermore, extrudability, which characterizes the ability of materials to be continuously extruded through a nozzle while maintaining geometric stability and minimizing blockage and segregation [29], is more related to dynamic yield stress and plastic viscosity [28,29]. According to Si et al. [29], buildability is governed by static yield stress (the minimum shear stress required to initiate flow) and thixotropy, in addition to the geometry of the concrete structure [28,29].

Thixotropy and structural build-up are, therefore, critical for 3D printing applications because they govern the ability of the material to recover stiffness after extrusion. Previous studies have shown that rapid structural rebuilding significantly improves buildability and layer stability in extrusion-based 3D printing systems [20,32]. Consequently, the rheological behavior of printable materials requires an appropriate balance between flowability during pumping and extrusion and structural recovery after deposition. The specific rheological requirements, structural build-up characteristics, and optimization strategies associated with 3DCP permanent formwork systems are discussed in Section 4.1.

### 3.3. Concrete Material Components in 3DCP Permanent Formworks

According to recent literature, a variety of cementitious composites, such as printed concretes, engineered cementitious composites (ECCs), strain-hardening cementitious composites (SHCC), and fiber-reinforced cementitious materials, have been utilized in 3D concrete printing of stay-in-place formworks. Table 1 presents the available literature and the related concrete materials used for 3DCP permanent formworks. The available literature is strongly dominated by OPC and OPC-supplementary cementitious material (OPC-SCM)-based binders, while direct investigations of alternative binder systems remain very limited. In particular, geopolymer-based 3D-printed permanent formwork has been demonstrated in only a small number of studies, in contrast to the considerably broader literature on geopolymer materials developed for general 3D concrete printing applications. SCMs commonly incorporated into 3DCP formworks include silica fume, fly ash, slag, and limestone powder to improve sustainability, rheology, buildability, extrusion stability, and long-term performance. Related 3D printing studies have likewise demonstrated the printability benefits of highly reactive additions, such as metakaolin and silica fume [33]. For instance, Zhu et al. [34] investigated 3D-printed permanent concrete formworks for reinforced concrete columns using printable cementitious mixtures containing OPC, silica fume, sulfoaluminate cement (SAC), fine sand, a viscosity-modifying admixture, and a polycarboxylate superplasticizer. The authors used silica fume and a small amount of SAC (3%) in all their compositions to improve the hydration reaction and early-age structural build-up of the printed shells. Similarly, in studies involving ECC- and SHCC-based permanent formworks, Bai et al. [35] and Singh et al. [36] investigated 3D-printed ECC and SHCC permanent formworks and reported the use of fly ash and silica fume as SCMs to improve ductility and crack control. The addition of viscosity-modifying agents (VMAs), such as hydroxypropyl methyl cellulose (HPMC), is also common in 3DCP formwork mixtures. In particular, VMAs were found to enhance printability, shape retention, and dimensional stability, although HPMC was also reported to cause some reduction in the compressive and flexural strengths of the shell material [34]. Importantly, dedicated studies on shrinkage-reducing admixtures in permanent formwork applications are limited, while related 3D concrete printing investigations have identified differential deformation between the printed shell and cast concrete core as a critical issue in printed formwork systems [37].

Unlike OPC-based systems, the use of geopolymer-based printable materials for permanent formwork applications remains largely unexplored, despite the extensive research devoted to geopolymer materials in the broader field of 3D concrete printing [44]. Therefore, the evidence discussed in this review is distinguished between direct experimental investigations of geopolymer permanent formwork and supporting knowledge derived from general 3D-printed geopolymer studies. In the permanent formwork literature identified in this review, Bong et al. [45] used a 3D-printed engineered strain-hardening geopolymer composite (3DP-EGC) as permanent formwork for a reinforced concrete beam, providing direct experimental evidence of its application in a printed-cast structural system. By contrast, studies on waste-derived geopolymer binders using brick and processed glass wastes [46,47,48] and broader investigations of alkali-activated materials and geopolymer precursors and activators [49,50] concern material development rather than permanent formwork applications. Their findings are, therefore, considered here only as supporting knowledge for the potential development of future geopolymer permanent formwork systems.

Beyond OPC and geopolymers, other lower-clinker or alternative cement systems may also be relevant to future printable permanent formworks; however, the available evidence is primarily derived from general material-level studies rather than direct permanent formwork demonstrations. Studies on ternary binders for 3D printing show that early-age rheology and hydration can be deliberately controlled to meet printing requirements [51]. For calcium aluminate cement (CAC), fly ash has been shown to modify the fresh properties, hydrate conversion, and strength development [52]. Limestone calcined clay cement (LC3) can reduce clinker content to about 50% while maintaining comparable mechanical properties and improving some durability aspects [53]. Calcium sulfoaluminate (CSA) cement has also been investigated as a lower-CO_2_ alternative to Portland cement [54] and as an alternative cement system for waste encapsulation [55]. In the specific context of 3D printing, the fresh and rheological properties of LC3 concrete are sensitive to superplasticizer addition time [56]. Although these studies establish the material-level relevance of CAC, CSA, and LC3 to 3DCP technology, they should not be interpreted as direct evidence of their performance as permanent formwork. Their use as primary binders in 3DCP stay-in-place formwork systems remains largely unexplored. These alternative binders may offer potential advantages, including reduced embodied carbon, rapid strength development, improved durability, and enhanced rheological control; however, their interfacial bonding with post-cast concrete, dimensional stability, shrinkage compatibility, reinforcement integration, and long-term durability still require direct experimental investigation.

Rapid structurization depends on how quickly the fresh material stiffens after deposition. Beyond binder systems, the currently available studies on 3D-printed concrete permanent formworks have also investigated the influence of aggregates, fibers, and reinforcement strategies on the performance of composite members. Most printable mixtures were developed with fine aggregates to avoid nozzle blockage and improve extrusion quality [34]. However, some studies have successfully introduced coarse aggregates into permanent formwork systems [39]. Fiber reinforcement has also been increasingly investigated in permanent formwork systems. The fibers incorporated have primarily been polyethylene (PE) and polyvinyl alcohol (PVA) fibers, which improve crack resistance, ductility, and durability of stay-in-place systems [35,40]. Likewise, Liu et al. [40] proposed a hybrid approach combining PVA fibers with basalt fiber-reinforced polymer bars in permanent formworks and reported improvements in the structural behavior and interfacial bonding between the printed shell and cast concrete core. Similar to binder systems, the available literature on different aggregate and fiber types remains limited, particularly with respect to comparative investigations.

## 4. Properties of 3DCP Permanent Formwork Systems

Based on the available literature, the performance of 3DCP permanent formwork can be interpreted through an interconnected sequence linking material composition, printing process, shell characteristics, and composite structural response. Material composition governs rheological behavior, structural build-up, and early-age mechanical development, which in turn determine the feasible printing parameters and the ability of the deposited material to retain its geometry. The interaction between rheology and printing parameters controls filament dimensions, layer geometry, surface morphology, interlayer integrity, and dimensional accuracy of the printed shell. These characteristics subsequently govern the interaction between the printed formwork and post-cast concrete through mechanical interlocking, interfacial adhesion, moisture exchange, and dimensional compatibility. Ultimately, the combined effects of shell material properties, geometry, printing-induced anisotropy, and shell–core interface quality determine stress transfer, confinement, cracking behavior, ductility, and the load-bearing contribution of the printed formwork. Accordingly, the following sections discuss fresh-state behavior, printing parameters, mechanical performance, durability, and microstructure as interconnected aspects of the overall performance of 3DCP permanent formwork systems.

### 4.1. Fresh-State and Rheological Properties of 3DCP Permanent Formwork

The fresh-state performance of 3DCP permanent formwork is strongly dependent on the rheological properties discussed previously. Among the rheological parameters, yield stress is considered one of the most important properties influencing buildability and shape stability. However, the literature reports considerable variability in the static and dynamic yield stress values associated with suitable printability. These reported values should be interpreted with caution because measured rheological parameters depend not only on the material composition but also on the rheometer type and geometry, testing protocol, applied shear history, resting time, and constitutive model used to derive the rheological parameters [21,57]. Consequently, numerical values obtained using different rheometers or testing procedures are not necessarily directly comparable, and the variability reported in the literature may reflect both actual differences in material behavior and differences arising from the measurement methodology. For example, yield stress values ranging from approximately 0.9 to 5.3 kPa were reported using the ICAR rheometer, whereas values of about 1.72–1.84 kPa were obtained using the RotoVisco RV1 and Brookfield rheometers [57]. These values are, therefore, presented to illustrate the range reported in the literature rather than to indicate direct differences in material performance. Excessively high yield stress may negatively affect pumpability and extrudability by increasing pumping pressure and reducing the continuity of material flow through the nozzle. Conversely, low yield stress may improve flowability but can result in filament spreading, instability, and poor dimensional accuracy, particularly in thin-walled permanent formwork systems [34]. For instance, according to Roussel [20], during the printing of a typical wall with a height of approximately 2.5 m, the yield stress in the bottom layers may need to reach values around 30 kPa to prevent structural collapse during layer-by-layer deposition. Similarly, static yield stresses of 0.273–1.132 kPa have been reported for fiber-containing mixtures using a Brookfield RST-SST rheometer [58]; however, these values should be interpreted within the specific testing conditions rather than directly compared with values obtained using other rheometers.

In addition, plastic viscosity strongly influences the pumping and extrusion behavior of printable mixtures. Reported plastic viscosity values for extrusion-based printable concretes range from approximately 16 to 46 Pa·s using the Viskomat XL rheometer (Schleibinger Geräte Teubert u. Greim GmbH, Buchbach, Germany) and from 1.7 to 21 Pa·s using other rheometers, depending on the printing system and mixture design [57]. As with yield stress, these ranges are method-dependent and should not be interpreted as directly comparable values across different rheometers and testing protocols. Mixtures with excessively high viscosity generally require higher pumping energy and may lead to nozzle blockage, discontinuous extrusion, and poor surface finish. In contrast, very low viscosity can cause segregation, bleeding, and inadequate shape retention after deposition [29].

Beyond these rheological requirements, the stability of successively deposited layers is governed by the evolving stiffness and strength of the fresh material relative to the increasing self-weight of the printed structure. Suiker et al. [59] identified two principal structural failure mechanisms during 3D concrete printing: elastic buckling, associated with insufficient stiffness and structural stability, and plastic collapse, which occurs when the developing stresses exceed the evolving strength of the fresh material (Figure 3). These failure mechanisms emphasize the importance of adequate early-age structural build-up for maintaining geometric stability as the printing height increases. Thixotropy and structural build-up are, therefore, critical for 3D printing applications because they govern the ability of the material to recover stiffness after extrusion. Previous studies have shown that rapid structural rebuilding significantly improves buildability and layer stability in extrusion-based 3D printing systems [20,32]. Structural build-up patterns reported for printable materials mainly vary depending on the mixture compositions (Table 2) [60].

Rapid structuration enhances the ability of printed layers to support additional layers and resist hydrostatic pressure during hybrid casting operations, which is particularly important for permanent stay-in-place formwork systems. However, excessively rapid structuration may reduce interlayer bonding quality because previously deposited layers become too stiff to form adequate interfacial adhesion with subsequent layers. Consequently, the successful implementation of sustainable printable materials in permanent formwork applications requires careful rheological control to ensure both printability and structural stability. Compared to the general 3DCP technology, only a limited number of studies have considered the fresh-state characterization of 3D concrete printing intended for permanent or stay-in-place formwork. By varying the HPMC dosage and the water-to-binder ratio, Zhu et al. [34] provided one of the most explicit fresh properties investigations, where the printable formwork was optimized through rheological testing, of static yield stress, thixotropy, structural build-up assessment, and green strength measurements. They showed that the optimized mixture containing 0.0006 HPMC by binder mass and a W/B ratio of 0.27, which exhibited the highest static yield stress, strongest thixotropic response, and a slump below 15 mm, achieved the maximum green strength, thereby improving the dimensional stability of the printed layers and their resistance to plastic deformation under self-weight and subsequent layer deposition, although failure still occurred once the critical layer limit was exceeded (Figure 4). In later studies, Kim et al. [61,62] expanded the application toward beams and composite columns, and the rheological properties of the printable material were evaluated as reference indicators for quality assessment and buildability prediction. According to these authors, under their adopted rheological testing conditions, the material exhibited a dynamic yield shear stress of 1356.1 Pa, a re-flocculation rate of 12.3 Pa/s, an initial static yield shear stress of 2832.1 Pa, and a structuration rate of 1.3 Pa/s, indicating pronounced thixotropy and progressive structural build-up suitable for the investigated buildability requirements. They further suggested using a slug-test apparatus, where the mass of freely extruded material droplets was measured to estimate the yield stress and, therefore, pumpability and extrudability of the printable mixture. It is important to note that most of the other studies on 3DCP permanent concrete formworks provided only limited consideration of fresh-state behavior, often without clear measurements of fresh properties. This highlights the need for further research on the relationships between rheology, printability, layer stability, deformation, open time, and structural performance to ensure reliable and scalable implementation of 3D-printed permanent formwork systems.

**Table 2:** Principal factors influencing the thixotropic structural build-up of cement-based materials relevant to 3D concrete printing. Adapted from Jiao et al. [60] under the CC BY 4.0 license.

Factors	Effect on Structural Build-Up
Increase in water-to-binder ratio	Decrease
Fly ash	Decrease (rarely increase)
Slag, silica fume, metakaolin	Increase
Superplasticizer	Decrease
Viscosity-modifying admixture (cellulose ether, nano-clay, etc.)	Increase
Accelerator	Increase
Retarder	Decrease
Aggregates or stiff fibers	Increase
Elevated temperature	Increase

**Figure 4:**
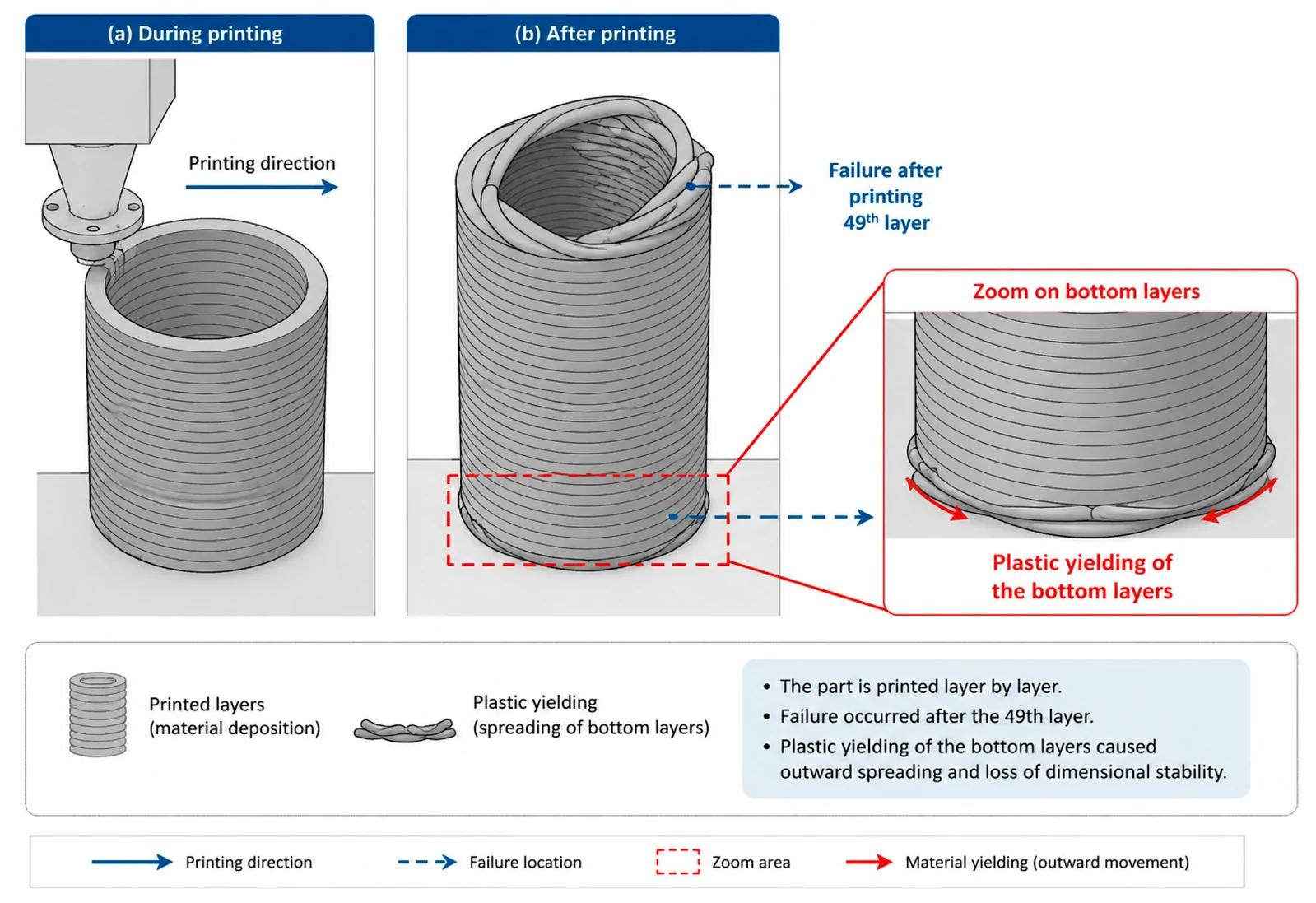
Schematic illustration of the buildability failure observed during fabrication of 3D-printed permanent concrete column formwork, showing plastic yielding and outward spreading of the bottom layers after the critical printing height was exceeded. Illustration prepared based on the experimental observations reported by Zhu et al. [34].

### 4.2. Printing Parameters of 3DCP Permanent Formwork

As for fresh-state rheological characterization, printability-related parameters associated with the actual fabrication of 3D-printed permanent formworks remain insufficiently investigated. Existing studies mainly reported practical printing parameters, such as nozzle diameter, nozzle speed, stand-off distance, layer height, flow rate, and printed geometry, to demonstrate the fabrication feasibility of beams, columns, and composite members. However, limited attention has been given to understanding how these parameters influence dimensional stability, layer deformation, geometric accuracy, interlayer consistency, surface quality, reinforcement accommodation, and the long-term composite interaction between the printed shell and cast concrete. These parameters are critical because they directly affect filament quality, layer deformation, dimensional stability, printable height, interlayer interfaces, and the final structural performance of the composite member. Table 3 summarizes the printing properties reported in the literature. Among the available studies, Kim et al. [61] evaluated nozzle diameters of 22 mm, nozzle speeds ranging from 4000 to 5000 mm/min, nozzle heights between 8 and 12 mm, and flow rates from 1.8 to 8.4 L/min. The greatest printable height and strengths were achieved at printing conditions of 4500/8/2.4 (speed/nozzle height/flow rate), which were selected for their printed formworks. In another study, Kim et al. [62] reported a nozzle diameter of 22.5 mm, stand-off distance of 6 mm, and printing speed of 1800 mm/min for reinforced concrete beam formworks. They further showed that extrusion pressure increased from 18.22 kPa at 15 min to 36.91 kPa after 65 min, while extrusion continuity decreased by 27.97%, demonstrating the significant influence of printing time on process stability and printability. Other studies, such as Zhu et al. [34], Guan et al. [63], Chen et al. [39], and Bang et al. [64], mainly reported practical printing parameters, such as nozzle diameter, nozzle speed, stand-off distance, layer height/width, extrusion rate, and printed geometry, used for ensuring appropriate filament deposition and dimensional stability of their 3DCP formworks. For instance, Guan et al. [63] used a layer width of 22 mm and a printing speed at 40 mm/s, while a total of 20 layers were printed. Future studies should, therefore, move beyond simple reporting of printing settings and establish standardized printability–performance relationships specifically for permanent formwork applications. Particular attention should be directed toward quantifying the influence of printing parameters on shell stability during casting, interfacial bonding with cast concrete, dimensional tolerances, anisotropic behavior, defect formation, and scalability for full structural components. In addition, the development of predictive models integrating rheology, printing parameters, and structural response would significantly contribute to the reliable design and industrial implementation of 3DCP permanent formwork systems.

**Table 3:** Printing parameters of 3DCP permanent formworks.

Study	Printing Method	Nozzle and Layer Characteristics	Printing Parameters	Time-Related Parameter	Formwork Configuration
Zhu et al. [34]	Extrusion-based 3DCP	Nozzle: 20 mm; layer height: 12 mm	Not clearly reported	Material rested 10 min before extrusion	Formwork thickness: 25 mm
Kim et al. [61]	3-axis extrusion-based 3DCP	Round nozzle: 22 mm; effective printed width: 31.59–32.68 mm	4500 mm/min/8 mm/2.4 L/min (printing speed/nozzle height/flow rate)	Not reported	Permanent formwork geometry evaluated through layer deformation and sharp-corner constraints
Kim et al. [62]	Extrusion-based 3DCP	Nozzle: 22.5 mm; 10-layer vertical deposition test	Printing speed: 1800 mm/min	Cement–water contact time < 1 h	RC beam permanent formwork
Guan et al. [63]	Extrusion-based 3DCP	Layer width: 22 mm; 20 layers	Printing speed: 40 mm/s	Not reported	2 bottom + 18 side layers; cover ≥ 30 mm; final dimensions within 5% of design
Bekaert et al. [65]	Extrusion-based 3D-printed stay-in-place formwork	Circular nozzle: 25 mm; free or compressed outflow using side trowels; layer height: 10 mm	Not reported	Printing interval: max. 12 s (Sequence 1); negligible (Sequence 2)	Rectangular formwork; 6 or 14 layers; nominal cover width: 40 mm
Bekaert et al. [66]	3D-printed stay-in-place formwork	Circular nozzle: 25 mm	Printing speed: 140 mm/s; stand-off distance: 20 mm	Not reported	Circular formwork; 450 mm cylindrical formwork filled with SCC
Bong et al. [45]	Gantry-type extrusion-based 3DCP	Circular nozzle: 20 mm	Printing speed: 25 mm/s; extrusion rate: ~0.75 L/min	Fresh concrete cast into 3DP-EGC formwork	Permanent RC beam formwork; two soffit printing patterns; prismatic geometry: 1800 × 200 mm
Wang et al. [38]	Extrusion-based 3DCP	Nozzles: 18, 30, and 38 mm; layer heights: 10, 15, and 20 mm	Not reported	Shell–core casting interval: 1, 3, 7, and 14 days	Printed section width: 100 mm
Bang et al. [64]	3D-printed permanent formwork for hybrid RC columns	Layer thickness: 20 mm; filament width: 40 mm	Travel speed: 5000 mm/min	Optimal casting window investigated	Rectangular formwork: 340 mm; cover thickness: 70 mm; 50 layers

Abbreviations: 3DCP, three-dimensional concrete printing; RC, reinforced concrete; SCC, self-compacting concrete; 3DP-EGC, 3D-printed engineered strain-hardening geopolymer composite.

### 4.3. Mechanical Properties of 3DCP Permanent Formwork Systems

Table 4 summarizes the structural configurations, specimen details, investigated mechanical properties, testing methods, and principal findings reported for 3DCP permanent formwork systems.

#### 4.3.1. Axial Compressive Properties

The current literature on 3D concrete printing permanent formworks has mainly focused on evaluating the axial behavior of composite concrete columns, where the printed shell remains in place and interacts structurally with the cast concrete core and reinforcement. The testing mostly included pure axial compression, ultimate bearing capacity, load-displacement behavior, stiffness, deformation capacity, confinement effect, interface performance under compression, and failure mode. Available investigations generally indicate that incorporating a 3D-printed permanent shell can modify and, in many configurations, improve the axial response of composite columns. However, these improvements should not necessarily be attributed exclusively to a confinement effect or to the use of 3D-printed formwork itself, because the investigated printed and reference specimens frequently differ in several interacting parameters. The structural response may result from the additional load-bearing cross-section provided by the shell, the mechanical properties of the printable material, passive confinement of the core, shell–core composite action and mechanical interlocking, as well as the amount and configuration of reinforcement. The relative contribution of these mechanisms has generally not been isolated experimentally. Consequently, comparisons with conventional cast specimens should be interpreted as the response of the complete printed shell–core composite system rather than as a direct measure of the structural benefit attributable solely to 3D printing.

For example, Zhu et al. [34] investigated circular reinforced concrete columns fabricated using 3D-printed permanent formwork filled with cast-in-place normal concrete under axial compression. The composite columns exhibited higher load-bearing capacity and improved structural integrity compared with conventional columns. In this case, the observed response was associated with several concurrent factors, including the higher compressive strength of the printed shell (40 MPa) relative to the core concrete (30 MPa), the additional shell cross-section, and the confinement provided by the formwork. Increasing the longitudinal reinforcement ratio up to 2.5% further enhanced stiffness and load capacity, while the concave–convex interface improved bonding and load transfer without peeling failure at the printed interfaces. Liu et al. [40] further evaluated circular columns incorporating 3D-printed PVA fiber-reinforced permanent formwork under both axial and eccentric compression. The composite columns demonstrated higher load-bearing capacity and lower displacement due to the synergistic interaction between the high-strength printed shell (61.2 MPa) and the cast concrete core (34.0 MPa). However, under eccentric loading, interlayer cracking reduced the load capacity of the specimen with the lower reinforcement ratio, although most composite columns still showed more than 11% improvement in bearing capacity. Complementary evidence on the axial behavior of hybrid printed-cast systems was reported by Giwa et al. [71], who investigated circular elements incorporating 3D-printed concrete shells with cast-in-place infill materials. Their results showed that the axial compressive performance of these hybrid elements was strongly influenced by the interaction and compatibility between the printed shell and the infilled core. The printed shell contributed to the overall load-bearing response, while differences in material properties and interfacial interaction affected the composite behavior, further highlighting the importance of effective shell–core interaction in the potential application of 3D-printed shells as permanent formwork (Figure 5). The recent study by Bang et al. [64], which investigated hybrid reinforced concrete columns incorporating 3D-printed formworks, demonstrated that the structural contribution of the printed shell strongly depends on the casting window and interface quality. They also reported that hybrid columns exhibited higher peak loads and energy absorption than conventional reinforced concrete columns, while the printed shell provided effective passive confinement under core-focused loading. In contrast to the findings of most studies on 3DCP permanent formwork, Bates and Aslani [72], who investigated 3D-printed concrete columns reinforced with GFRP rebars, reported that the printed columns exhibited approximately 50% of the peak load capacity of conventionally mold-cast columns, mainly due to weak interlayer bonding and delamination between printed layers, highlighting the need for further research aimed at improving the interlayer integrity and enhancing the axial performance of the printed column systems. In addition, the current literature remains limited regarding the quantification of confinement efficiency and the influence of key parameters, such as shell thickness, printing orientation, interlayer anisotropy, reinforcement configuration, and printing-induced defects, on the axial behavior of composite columns. Overall, the available studies demonstrate the structural potential of printed shell–core systems, but do not yet permit the individual contribution of the printed shell to be fully quantified. Future investigations should, therefore, employ controlled reference specimens that independently vary shell thickness and cross-section, shell material strength, interface geometry, and reinforcement configuration. Such comparisons would allow the contributions of direct shell load resistance, passive confinement, mechanical interlocking, reinforcement, and composite shell–core interaction to be separated and quantified.

#### 4.3.2. Tensile and Flexural Properties

The tensile and flexural behavior of 3DCP permanent formwork systems has mainly been investigated for reinforced concrete beams incorporating printed shells acting as stay-in-place tensile reinforcement layers. Existing studies generally indicate that the printed formwork can act not only as a permanent mold but also as an active component of the composite member. However, the observed flexural response reflects the combined effects of the printed-shell material properties, additional shell cross-section, reinforcement configuration, printing orientation, shell geometry, and interfacial bonding rather than the presence of the printed formwork alone. Permanent formworks fabricated using engineered strain-hardening geopolymer composites demonstrated superior flexural performance compared with conventional mold-cast reinforced concrete beams by enabling a deflection-hardening response after steel yielding instead of the typical deflection-softening behavior [45]. Kim et al. [62] further demonstrated the structural contribution of 3D-printed permanent formwork under flexural loading. The beams incorporating 3DPF reached ultimate loads of 148.42–149.23 kN, comparable to the 153.67 kN of the corresponding cast-in-place beam. However, the printed-formwork beams exhibited lower cracking loads and deformation capacity, highlighting the influence of printing-induced anisotropy and interlayer bonding on crack development and ductility [62].

Guan et al. [63] showed that the interface configuration significantly influenced the flexural response of beams with 3D-printed permanent formwork (Figure 6). In particular, the bottom-rib configuration enhanced composite action and load transfer, increasing the bending capacity by 14.3% compared with the completely cast reference beam [63]. Raza et al. [73] investigated the flexural behavior of load-bearing 3D-printed concrete stay-in-place formwork under four-point bending. The specimens exhibited progressive damage from vertical flexural cracking to web-shear cracking and ultimately horizontal shear failure (Figure 7), highlighting the influence of shell geometry and printing-induced discontinuities on flexural performance. Collectively, these findings confirm the strong influence of the mechanical and tensile properties of the printable material, particularly its post-cracking deformation and crack-control capability, on the structural contribution of 3DCP permanent formworks. In addition, the orientation of the printed layers significantly affected cracking resistance, where filament alignment parallel to the tensile stress promoted the formation of multiple fine cracks with smaller crack widths instead of localized wide cracks [45]. The geometric configuration of the printed shell also played a critical role in the structural response. Anchor-interface formwork configurations improved the shear-bearing capacity by up to 81.4% compared with conventional reinforced concrete members, while maintaining dimensional deviations within 5% of the design geometry [63]. Similarly, interfacial tensile and shear performance were highly dependent on the printing layer geometry and interface texture. A 15 mm printing layer height provided the optimum mechanical interlocking effect, whereas full recycled sand replacement reduced splitting tensile and shear strengths by 36.1% and 35.8%, respectively [69]. Physical grooving at the interface further enhanced tensile and shear bond performance by improving the contact area and mechanical interlocking between the printed shell and post-cast concrete [67].

#### 4.3.3. Interfacial Bonding Performance

The interfacial bond between 3D-printed permanent formwork and post-cast concrete governs stress transfer and composite action. Unlike general 3DCP elements, the printed shell must first withstand the lateral pressure exerted during casting of the fresh core concrete and subsequently remain bonded to the hardened core as an integral part of the composite member. This permanent shell–core interaction introduces additional requirements related to dimensional compatibility and time-dependent interfacial stresses. Existing studies have primarily characterized this interface through splitting tensile and shear tests, with surface geometry, casting interval, printed-material composition, and post-cast concrete properties identified as the main governing parameters. Overall, interfacial performance is governed by the coupled effects of surface morphology, casting interval, moisture exchange, printed-material properties, post-cast concrete composition, and curing conditions. Physical modification of the printed surface generally improves bonding through increased contact area and mechanical interlocking. Yu et al. [67] compared untreated, cement-paste-coated, grooved, and combined grooved–cement-paste interfaces. At a one-day casting interval, grooving increased the splitting tensile strength from 2.8 to 3.1 MPa. The physical treatment also increased shear strength by 30.7% relative to the corresponding control at a one-day interval. Conversely, combining grooves with cement paste produced variable and, in some cases, reduced bond performance because the paste partially obstructed the interlocking geometry and increased interfacial porosity. Bond strengths were generally higher at the one-day interval than at three days in this study [67]. The beneficial effect of surface geometry was also demonstrated for 3DP ultra-high-performance (UHP)-SHCC permanent formwork, where the printed shell was cured for 28 days before the normal concrete (NC) was cast. Grooved and as-printed interfaces produced splitting tensile bond strengths 92–115% higher than a smooth interface, whereas the smooth interface reached only 42% of the strength of monolithically cast normal concrete. This study confirmed that both deliberately introduced grooves and the intrinsic corrugation of printed layers can substantially increase the effective contact area and mechanical interlocking between the 3D-printed formwork and cast NC, resulting in improved interfacial bond performance [70]. The importance of interfacial integrity was also demonstrated by Giwa et al. [71] for hybrid elements incorporating 3D-printed cementitious shells as permanent formwork. The interaction between the printed shell and subsequently placed core material contributed to the composite response, while splitting tensile testing revealed interfacial debonding between the two materials (Figure 8). These observations further highlight the importance of adequate shell–core compatibility and interfacial adhesion for effective composite action in 3DCP permanent formwork systems.

Conflicting results have been reported regarding the effect of the casting interval on the interfacial bonding behavior between 3D-printed shell and cast-in-place concrete. Wang et al. [38] examined printed layer heights of 10, 15, and 20 mm and casting intervals of 1, 3, 7, and 14 days. The 20 mm layer height provided the highest splitting tensile and shear bond performance and casting the core concrete seven days after printing was recommended. The reported maximum shear strength was 2.8 MPa for the 20 mm configuration at seven days, representing a 91.8% increase relative to the corresponding one-day specimen. The results were associated with the combined effects of printed-surface roughness, mechanical interlocking, moisture exchange, and interfacial microstructure [38]. In contrast to Wang et al. [38], who obtained the highest performance at seven days, Yu et al. [67] observed lower bond strengths at three days than at one day. These apparently conflicting results suggest that casting interval should not be considered as an independent governing parameter, but rather as an indicator of the evolving condition of the printed substrate at the time of casting. The differences between the two studies may reflect the combined influence of printed-material composition, age-dependent hydration and stiffness development, moisture condition and curing environment, surface geometry, interface treatment, and the properties of the post-cast concrete. The optimum interval may correspond to a balance between hydration and stiffness development of the printed shell, which improves its mechanical integrity, and the progressive reduction in surface moisture and interfacial interaction as the substrate ages. Surface morphology further modifies this balance because pronounced layer corrugations or deliberately introduced grooves can enhance mechanical interlocking even when physicochemical interaction with the post-cast material is reduced. Consequently, different combinations of substrate maturity, moisture condition, surface characteristics, and post-cast concrete properties may shift the casting interval associated with the highest measured bond strength. Future studies should, therefore, relate casting time to measurable substrate properties, such as moisture condition, degree of hydration, matrix stiffness, and surface characteristics, rather than treating elapsed time alone as the governing parameter.

The composition and grading of the post-cast concrete also affect interface resistance. Chen et al. [74] reported that, for coarse-aggregate 3D-printed permanent formwork, increasing the proportion of smaller coarse aggregate in the post-cast concrete improved filling of the interface region and increased splitting tensile bond strength. Wang et al. [68] compared ordinary aggregate concrete, self-compacting concrete, self-compacting concrete containing an expansion agent, and epoxy-treated self-compacting concrete. The expansion-agent mixture provided the strongest interface, consistent with improved dimensional compatibility and reduced shrinkage separation. This dimensional compatibility is particularly critical for permanent formwork because the printed shell remains bonded to the post-cast concrete and contributes to the composite response of the resulting member. Differences in the age, material composition, hydration development, and moisture conditions of the printed shell and post-cast concrete can result in differential deformation that is restrained at the interface. Bekaert et al. [37] experimentally demonstrated the importance of this issue by showing that the curing history of 3D-printed formwork significantly affects the differential shrinkage and strain development of the combined printed-cast concrete element. Differential deformation associated with hydration-induced thermal effects, autogenous shrinkage, and drying shrinkage may generate tensile and shear stresses along the shell–core interface, potentially promoting cracking, interfacial debonding, and a reduction in effective composite action. Therefore, interfacial performance should be considered not only in terms of initial tensile and shear bond strengths, but also in relation to the dimensional compatibility and time-dependent deformation of the two materials. In contrast, epoxy treatment reduced splitting tensile strength by 65.1% relative to untreated self-compacting concrete, indicating that a continuous coating may interrupt direct contact and mechanical interlocking despite altering the fracture response [68]. The importance of interface morphology and mechanical interlocking is further demonstrated by the CT observations of Guan et al. [63], which revealed the mesoscale interaction between the printed formwork and cast concrete. Similarly, printable-material composition and layer geometry are also important. Bai et al. [69] reported a non-monotonic influence of fiber content and layer height, with a 15 mm layer height providing the most favorable interfacial performance among the investigated configurations. Complete replacement of natural sand with recycled sand reduced splitting tensile and shear bond strengths by 36.1% and 35.8%, respectively, demonstrating that changes to the printed matrix can influence interface strength through their effects on material strength, surface formation, and interlocking quality [69]. It is worth noting that the interfacial bonding between geopolymer-based 3D-printed permanent formwork and conventional cast-in-place concrete remains largely unexplored, as no studies addressing this topic were identified in the current literature.

It is important to mention that, as summarized in Table 5, direct comparison of the reported bond strengths remains challenging because casting interval varies simultaneously with substrate composition, curing and moisture conditions, interface geometry, and post-cast concrete properties. The available results, therefore, support interpretation of interfacial performance through these coupled variables rather than through casting interval alone.

### 4.4. Durability Properties of 3DCP Permanent Formwork Systems

Durability remains one of the least investigated aspects of both cementitious and geopolymer 3D-printed concrete permanent formwork systems. To date, the available durability investigations have been limited exclusively to cementitious permanent formworks, whereas no durability studies have been reported for geopolymer permanent formwork systems. Among the available literature, Bekaert et al. [65,66] reported the only comprehensive durability assessment specifically evaluating 3D-printed cementitious permanent formwork acting as a protective concrete cover. The results demonstrated that a 3D-printed shell should not automatically be considered equivalent to conventionally cast concrete cover solely on the basis of nominal thickness, as its protective performance also depends on curing conditions, pore structure, filament compaction, surface finishing, and the continuity of transport pathways created during printing. In particular, preventing early-age drying by curing 3D-printed permanent formwork under a high-humidity environment (RH > 95%) significantly reduced porosity (Figure 9), water absorption, carbonation, and chloride migration compared with air-drying conditions (RH ≈ 60%). These findings also indicate that conventional durability requirements for concrete cover cannot yet be directly transferred to 3D-printed permanent formwork based solely on an equivalent nominal shell thickness. Unlike conventionally cast cover, the printed shell is inherently layered and may exhibit anisotropic transport behavior associated with interlayer interfaces, local variations in compaction, and printing-induced defects. Preferential ingress of water, chlorides, CO_2_, and other aggressive agents along weak or more porous interlayer regions could, therefore, reduce the effective protection provided to embedded reinforcement, even when the nominal shell thickness satisfies conventional cover requirements. This protection may be further compromised by cracking within the printed shell or debonding at the shell–core interface, which can create additional pathways for moisture and aggressive species toward the reinforced concrete core. However, direct experimental evidence linking these features to reinforcement corrosion in 3DCP permanent formwork systems remains unavailable. Overall, the durability of cementitious and geopolymer 3DCP permanent formwork systems remains largely unexplored. No studies were identified within the adopted search strategy that investigated the durability of geopolymer-based 3D-printed permanent formwork or directly compared its durability performance with cementitious systems. Furthermore, the available literature identified in the current review indicates that key durability aspects, including freeze–thaw resistance, de-icing salt scaling, sulfate and acid attack, reinforcement corrosion, abrasion, fire resistance, and wetting–drying cycles, remain insufficiently investigated in 3D-printed permanent formwork systems. These durability mechanisms are particularly relevant to bonded cementitious systems, where environmental exposure can affect not only the constituent materials but also the integrity of the interface and the resulting composite response [75,76,77,78]. In 3D-printed permanent formwork systems, this issue is especially important because the printed shell remains permanently bonded to the cast concrete and forms part of the external protective envelope of the composite member. Therefore, the effects of environmental exposure on the long-term integrity of the printed shell-cast concrete interface under realistic service conditions require systematic investigation. These knowledge gaps should be addressed before the widespread structural implementation of both cementitious and geopolymer 3DCP permanent formwork systems. In addition, the long-term performance of 3DCP permanent formwork under actual field exposure conditions requires further investigation. More recently, Shiotani et al. [79] extended durability-related assessment to an actual bridge-pier footing constructed with 3DCP permanent formwork using visual inspection, hammering, and elastic-wave-based non-destructive testing. As illustrated in Figure 10, visual inspection revealed cracks up to 0.6 mm wide, while hammering identified a large region potentially affected by interlayer defects or delamination between the printed formwork and post-cast concrete. Subsequent NDT and borescope observations confirmed the ability to detect internal discontinuities and assess interfacial integrity. These findings highlight that long-term performance of 3DCP permanent formwork depends not only on material durability but also on maintaining the integrity of printed layers and their bond with the cast concrete, while demonstrating the potential of NDT for in-service condition assessment.

### 4.5. Microstructural Characterization

Microstructural characterization of the interface between 3D-printed permanent formwork and post-cast concrete remains limited but has provided valuable insights into the mechanisms governing interfacial bonding. The available microstructural investigations have been conducted almost exclusively on cementitious permanent formwork systems, whereas comparable studies on geopolymer permanent formworks have not yet been reported. Consequently, the current understanding of interface development is largely derived from cement-based printable materials. The limited available studies indicate that interface morphology, pore characteristics, and surface roughness are the principal microstructural features governing stress transfer and bond performance in 3DCP permanent formwork systems. Interfaces exhibiting more favorable interfacial characteristics and enhanced mechanical interlocking generally corresponded to higher splitting tensile and shear bond strengths, whereas interfaces containing greater interfacial defects or discontinuities were more susceptible to interfacial debonding [67,69]. CT observations further demonstrated that the mesoscale architecture of the printed formwork–cast concrete interface depends strongly on the adopted bonding configuration (Figure 11). Guan et al. [63] showed that interfacial bonding can be enhanced through increased surface roughness and mechanical interlocking between the printed shell and cast concrete, while shear connectors provided the most effective improvement in composite interaction. The available evidence further indicates that printing parameters and material composition significantly influence interface development. An optimum printing layer height produced the most favorable interfacial characteristics and the highest bond performance, whereas increasing the recycled-sand replacement ratio progressively deteriorated the interface, resulting in corresponding reductions in splitting tensile and shear bond strengths [69]. Similarly, modifying the interface geometry through physical grooving or utilizing the inherent as-printed surface texture increased the effective bonding area and enhanced mechanical interlocking, thereby improving the interfacial bond [67,70]. The evolution of the interfacial microstructure was also strongly influenced by the casting interval. A one-day interval resulted in the formation of a water film at the interface, reducing adhesion between the printed shell and the fresh concrete. In contrast, a seven-day interval produced the most compact interfacial microstructure, characterized by a large amount of ettringite (AFt) and a flocculent C-S-H gel, which effectively filled the interface gaps. Further delaying casting to 14 days introduced interfacial voids, which the authors attributed to excessive moisture exchange, resulting in reduced bond performance [38]. Overall, the microstructural characterization in these studies integrated X-CT, SEM-EDS, and three-dimensional surface metrology. However, these investigations have been almost exclusively limited to cementitious permanent formwork systems, while comparable studies on geopolymer permanent formworks, particularly those relating interface microstructure to transport properties, durability, and long-term structural performance, remain absent. Furthermore, quantitative relationships between interfacial microstructure, transport properties, and the long-term behavior of composite members have yet to be established, representing an important direction for future research.

## 5. Sustainability and Life-Cycle Assessment

The sustainability of 3D-printed permanent formwork systems has received very limited attention in the current literature. Although numerous studies have demonstrated the structural feasibility of cementitious and geopolymer permanent formworks, their environmental and economic performances have rarely been quantified. Nevertheless, eliminating conventional temporary formwork can provide sustainability advantages by reducing formwork-related material consumption, construction waste, and associated costs, depending on the construction scenario. Previous studies on 3D concrete printing have reported project cost reductions of up to 78%, labor savings approaching 60%, and formwork-related cost savings of up to 63% under specific construction scenarios [80]. In addition, 3DCP facilitates the utilization of locally available materials, thereby reducing transportation requirements, supply-chain constraints, and the associated environmental impacts. The environmental benefits are also associated with the incorporation of alternative binders and recycled materials, an approach widely investigated in sustainable 3D concrete printing [81]. Cementitious printable mixtures have increasingly incorporated supplementary cementitious materials, such as fly ash, ground-granulated blast-furnace slag, silica fume, and rice husk ash, while geopolymer systems completely replace OPC with alternative aluminosilicate precursors. Comparative life-cycle assessment has also been applied to construction- and demolition-waste-based geopolymer binders, supporting the need to evaluate environmental performance at the binder-system level [82,83]. These approaches have the potential to reduce clinker consumption and associated CO_2_ emissions while maintaining the rheological and mechanical requirements for extrusion-based printing. However, despite these advantages, the environmental performance of current 3DCP mixtures remains constrained by their relatively high binder content. Many printable cementitious mixtures employ relatively high binder contents, with some formulations containing more than 800 kg/m^3^ of OPC to satisfy pumpability, extrudability, and buildability requirements, resulting in high embodied CO_2_ emissions [78]. Consequently, the continued development of low-carbon printable binders and standardized mixture-design methodologies remains essential for improving the sustainability of 3DCP permanent formwork systems. The importance of material selection is further demonstrated by the dominance analysis of conventional and 3D-printed formwork scenarios reported by Albrecht et al. [84], which showed that the environmental impacts varied considerably according to the constituent materials and mixture composition, as illustrated in Figure 12. Therefore, the elimination of temporary formwork alone should not be interpreted as evidence of lower environmental impact. A comprehensive assessment should account for the production and quantity of the printable shell material, energy consumed during material preparation and printing, transportation, construction waste, and the number of reuse cycles of conventional formwork. For a cradle-to-grave assessment, the system boundary should additionally include potential differences in maintenance and service life, as well as end-of-life processes, including demolition, recycling, and disposal. The relative contributions of these factors are expected to depend strongly on material composition, structural geometry, construction method, and conventional-formwork reuse rate. Similarly, from a life-cycle perspective, the environmental benefits of 3DCP permanent formwork systems remain largely unexplored. Liu et al. [85] reported the only life-cycle assessment identified in the current literature comparing contour crafting permanent formwork with conventional cast-in-place concrete construction. The study demonstrated that the environmental performance of 3D-printed permanent formwork is strongly dependent on structural complexity and the reuse rate of conventional formwork. For simple and repetitive structural components, conventional construction generally exhibited a lower environmental impact because reusable steel or timber formwork distributed its environmental burden over multiple casting cycles. Under these conditions, the additional material required for the printed permanent formwork offset the environmental benefits associated with eliminating temporary formwork. In contrast, for complex and non-repetitive geometries requiring customized disposable formwork, such as freeform or saddle-shaped structures, 3D-printed permanent formwork became environmentally preferable, providing environmental impact reductions ranging from 5% to 64%, depending on the impact category considered [85]. These findings indicate that the environmental competitiveness of 3DCP permanent formwork increases with geometric complexity while decreasing as the reuse rate of conventional formwork increases. Accordingly, 3DCP permanent formwork appears most advantageous for geometrically complex, customized, and low-repetition structural elements for which conventional formwork requires extensive fabrication, specialized molds, or single-use formwork. Potential applications include freeform structural components, architecturally complex columns and walls, curved or variable-section members, and customized structural elements produced in small series. Conversely, simple and highly repetitive structural elements, for which conventional formwork can be efficiently reused over multiple casting cycles, are less likely to provide a strong environmental justification for 3DCP permanent formwork. Future sustainability assessments should, therefore, establish application-specific break-even conditions considering geometric complexity, conventional-formwork reuse rate, printed-shell material consumption, structural contribution of the shell, and potential service-life benefits, rather than considering 3DCP permanent formwork as a universally preferable alternative. Despite these promising findings, comprehensive life-cycle cost assessments (LCCAs) and cradle-to-grave life-cycle assessments (LCAs) are also lacking. Likewise, the potential environmental benefits associated with extended service life, reduced maintenance, improved durability, alternative printable binders, recycled constituents, and structural optimization have not yet been systematically quantified. These aspects represent important research priorities for establishing the true sustainability of 3DCP permanent formwork systems and supporting their broader implementation in sustainable construction.

## 6. Research Gaps and Future Perspectives

Despite the considerable progress achieved in the development of 3D-printed permanent formwork systems, several fundamental scientific and engineering challenges remain before their widespread implementation in structural engineering can be realized. The current literature is heavily dominated by cementitious permanent formwork systems, whereas geopolymer-based permanent formworks remain largely unexplored. Consequently, direct comparisons between cementitious and geopolymer systems regarding their fresh-state behavior, structural performance, interfacial bonding, durability, and sustainability are currently unavailable. To provide a more systematic roadmap for future development, the principal research needs are organized below according to the main material, process, structural, durability, implementation, and sustainability challenges.

- Materials, Rheology, and Printing Process

Future research should focus on establishing a comprehensive understanding of the advantages and limitations of cementitious and geopolymer material systems while exploring low-carbon binders, recycled constituents, and carbon-storing printable materials. Material development should consider the combined requirements of rheology, printability, mechanical performance, dimensional stability, and durability rather than optimizing these properties independently. Further studies are also required to establish quantitative relationships between printing parameters, including extrusion rate, printing speed, nozzle geometry, layer height, and interlayer time interval, and the resulting dimensional accuracy, surface characteristics, interlayer quality, and mechanical performance. Standardized rheological and printability characterization procedures would further facilitate meaningful comparison among different material and printing systems.

- Interface Engineering and Composite Action

Interfacial bonding continues to represent one of the most critical aspects governing the performance of permanent formwork systems. Although considerable progress has been made in understanding the influence of interface geometry, surface roughness, pore structure, printing parameters, and casting interval, contradictory conclusions regarding the optimum casting interval indicate that the governing mechanisms are still not fully understood. Future studies should establish quantitative relationships between moisture exchange, hydration development, interface morphology, mechanical interlocking, and bond performance. Particular attention should also be given to differential deformation between the printed shell and post-cast concrete and its influence on time-dependent interfacial stresses, cracking, and debonding. These investigations should be extended to geopolymer permanent formwork systems, where comparable studies remain largely unavailable.

- Structural Design, Modeling, and Reinforcement Integration

Although composite members incorporating printed permanent formwork have frequently exhibited improved structural response, the individual contribution of the printed shell to this improvement remains insufficiently understood. Existing studies have demonstrated enhanced axial load capacity, flexural performance, cracking resistance, and structural integrity; however, the mechanisms governing these improvements have not been fully quantified. In particular, the contributions of the shell material and cross-section, confinement, reinforcement, interface configuration, and composite shell–core interaction should be independently evaluated. The effects of shell thickness, printable material properties, reinforcement ratio and configuration, and printing orientation also require systematic investigation. Furthermore, current studies have focused primarily on columns and beams, while slabs, walls, beam-column joints, and more complex structural systems have received comparatively little attention. Analytical and numerical models and ultimately performance-based design methodologies should, therefore, be developed to account for the anisotropic nature of printed concrete, shell–core composite action, reinforcement contribution, confinement, and interfacial behavior.

- Durability and Service Life

Durability remains one of the least developed research areas. Apart from the durability assessment reported by Bekaert et al. [65,66], comprehensive investigations of the long-term performance of 3D-printed permanent formwork systems remain very limited. Future studies should address freeze–thaw cycles, de-icing salts, sulfate and acid attack, reinforcement corrosion, abrasion, fire exposure, wetting–drying cycles, and the long-term integrity of the printed shell-cast concrete interface under realistic service conditions. Particular attention should be given to directional transport through the layered printed shell, interlayer defects, cracking, shell–core debonding, and their potential implications for reinforcement protection. Long-term experimental data are ultimately required to support reliable service-life prediction for both cementitious and geopolymer permanent formwork systems.

- Scale-Up, Quality Control, and Standardization

Another important research need concerns the transition from laboratory-scale investigations to full-scale construction. Future studies should evaluate scale effects, lateral pressure during casting, geometric tolerances, printing variability and interruptions, construction joints, reinforcement placement, material consistency, and environmental variability under actual site conditions. Reliable quality-control and non-destructive inspection methods are also required to assess dimensional accuracy, layer integrity, cracking, and shell–core bonding. The bridge-pier footing investigated by Shiotani et al. [79], where cracking and regions potentially affected by interlayer defects or delamination were identified, demonstrates that laboratory feasibility does not necessarily ensure reliable field performance. Full-scale demonstrations and long-term field monitoring are, therefore, required to establish standardized testing procedures, acceptance criteria, and design provisions for practical implementation.

- Environmental and Economic Assessment

Sustainability assessments remain limited, with very few studies quantitatively evaluating the environmental performance of permanent formwork applications. Future studies should directly compare cementitious and geopolymer systems and evaluate the influence of alternative binders, recycled materials, printing energy, transportation, conventional-formwork reuse, structural optimization, maintenance, service life, and end-of-life scenarios. Comprehensive cradle-to-grave life-cycle assessments and life-cycle cost analyses are, therefore, required to establish the environmental and economic conditions under which 3DCP permanent formwork provides advantages over conventional construction. Particular attention should be given to application-specific break-even conditions associated with structural geometry, conventional-formwork reuse rate, printed-shell material consumption, and potential service-life benefits.

To facilitate the progressive development of these systems, the identified research needs can be considered across different implementation horizons. In the short term, priority should be given to standardized material, rheological, printability, dimensional, and interface characterization, and to establishing reproducible relationships between printing conditions and shell performance. In the medium term, research should focus on structural modeling, reinforcement integration, durability and reinforcement protection, full-scale validation, and reliable quality-control methods. In the long term, these developments should support performance-based design provisions, standardized acceptance criteria, validated service-life models, long-term field validation, and comprehensive environmental and economic assessment. Such a progression would facilitate the transition of 3DCP permanent formwork from experimental demonstrations toward reliable structural implementation.

## 7. Conclusions

This review provides a comprehensive assessment of the current state of knowledge on cementitious and geopolymer 3D-printed concrete permanent formwork systems, highlighting their structural potential, interfacial behavior, durability, sustainability, and future research needs. Based on the available literature, the following principal conclusions can be drawn:

- The 3D-printed permanent formwork has demonstrated significant potential as a stay-in-place structural system, providing construction advantages through the elimination of temporary formwork and, under appropriate material, interface, and structural configurations, contributing to the load-bearing response of reinforced concrete members.
- Composite members incorporating 3D-printed permanent formwork frequently exhibited improved axial load capacity, flexural behavior, cracking resistance, ductility, and structural integrity. However, these improvements were dependent on the investigated material properties, shell geometry, reinforcement configuration, printing quality, and shell–core interaction. Weak interlayer bonding, cracking, or delamination can reduce the structural contribution of the printed shell, indicating that improved performance should not be considered an inherent outcome of using 3D-printed permanent formwork.
- Interfacial bonding was identified as one of the most critical factors governing structural performance. The literature consistently demonstrates that interface geometry, surface roughness, pore characteristics, and mechanical interlocking strongly influence tensile and shear bond strengths. Physical surface treatments, optimized printing layer heights, and inherent as-printed surface textures generally provided the most effective bonding mechanisms, whereas epoxy- or cement-paste-based interface treatments did not consistently improve bond performance.
- The evolution of the printed shell-cast concrete interface remains insufficiently understood. Although moisture exchange, hydration development, and interface morphology have been shown to govern bond development, conflicting findings regarding the optimum casting interval indicate that the underlying mechanisms have not yet been fully established.
- Current research is heavily dominated by cementitious permanent formwork systems, whereas geopolymer permanent formworks remain in their early stages of development. Although the limited available studies indicate promising structural performance and potential sustainability benefits, comprehensive investigations addressing their structural behavior, interfacial bonding, durability, and long-term performance are still lacking.
- Durability remains one of the least developed research areas. Existing studies are almost exclusively limited to cementitious permanent formworks, with only one comprehensive investigation evaluating curing-dependent durability and service-life indicators. The long-term performance of both cementitious and geopolymer systems under realistic environmental exposure, including freeze–thaw cycles, de-icing salts, reinforcement corrosion, chemical attack, and fire, has yet to be established.
- The sustainability of 3DCP permanent formwork systems has not yet been comprehensively quantified. While the elimination of temporary formwork, the incorporation of supplementary cementitious materials and geopolymer binders, and the potential reduction in construction waste provide clear environmental advantages, dedicated life-cycle assessments, life-cycle cost analyses, and direct comparisons between cementitious and geopolymer systems remain very limited.
- Most available evidence is based on laboratory-scale studies, while field validation remains limited. Reported cracking, interlayer defects, and possible delamination highlight the need for full-scale validation, long-term monitoring, and reliable quality-control methods before broader structural implementation.
- The absence of standardized design methodologies represents one of the principal barriers to practical implementation. Design provisions accounting for the anisotropic characteristics of printed concrete, confinement effects, composite interaction, interfacial bond behavior, durability, and long-term structural performance are still unavailable and require systematic investigation.

Overall, the available evidence indicates that 3D-printed permanent formwork represents a promising approach for integrating digital fabrication with composite structural construction. However, the reported structural and sustainability benefits remain dependent on the material system, geometry, interface quality, printing conditions, and specific application. Future research should, therefore, move beyond demonstrating feasibility toward establishing performance-based design methodologies that integrate material optimization, structural mechanics, interfacial characterization, durability, sustainability, and life-cycle performance. Such developments, together with full-scale validation and long-term field monitoring, are essential before the safe and reliable broader implementation of 3D-printed permanent formwork systems in structural engineering practice can be established.

## Figures and Tables

**Figure 1:**
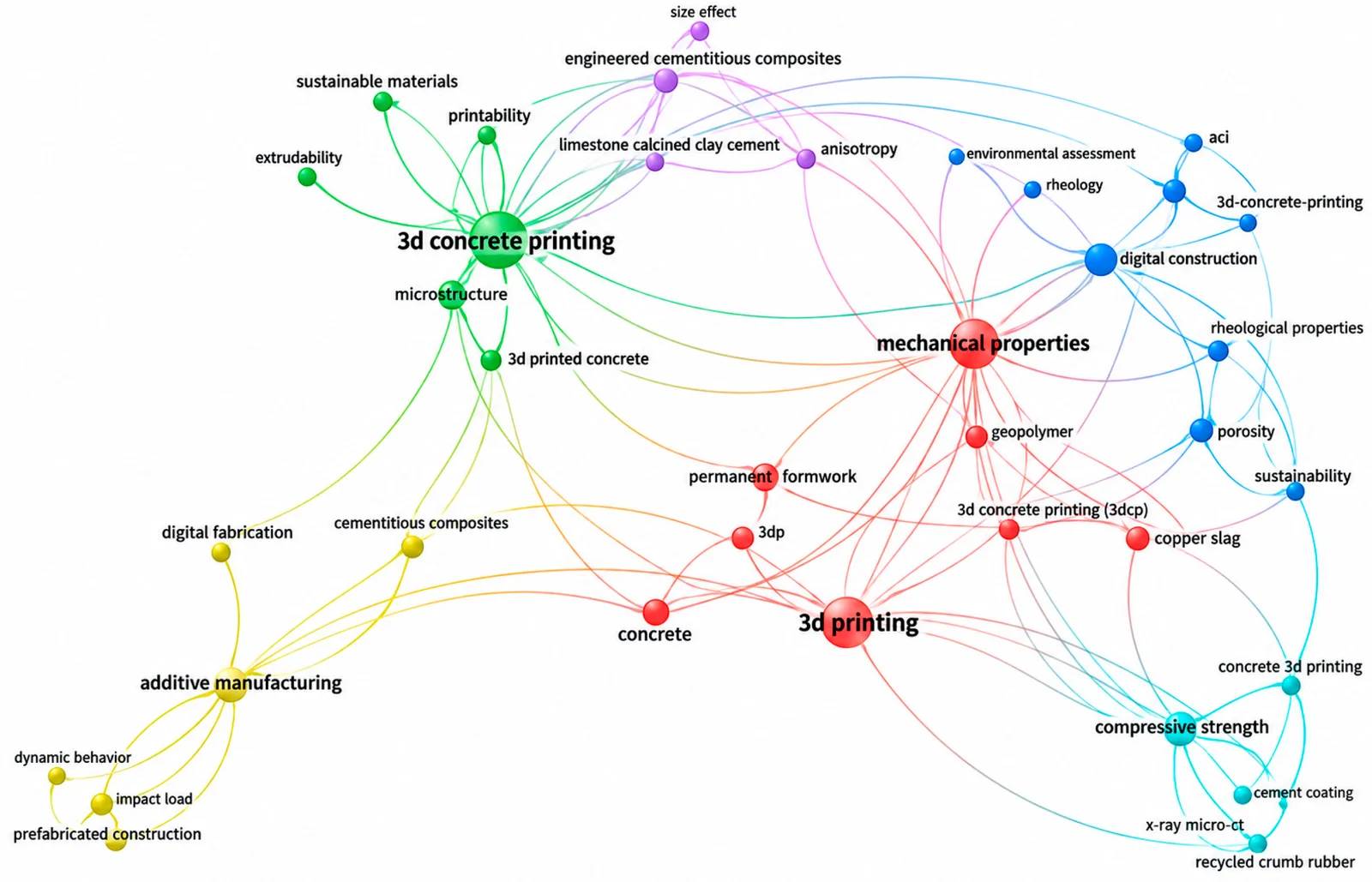
Network visualization of the keyword co-occurrence related to 3D-printed cementitious and geopolymer stay-in-place formworks.

**Figure 2:**
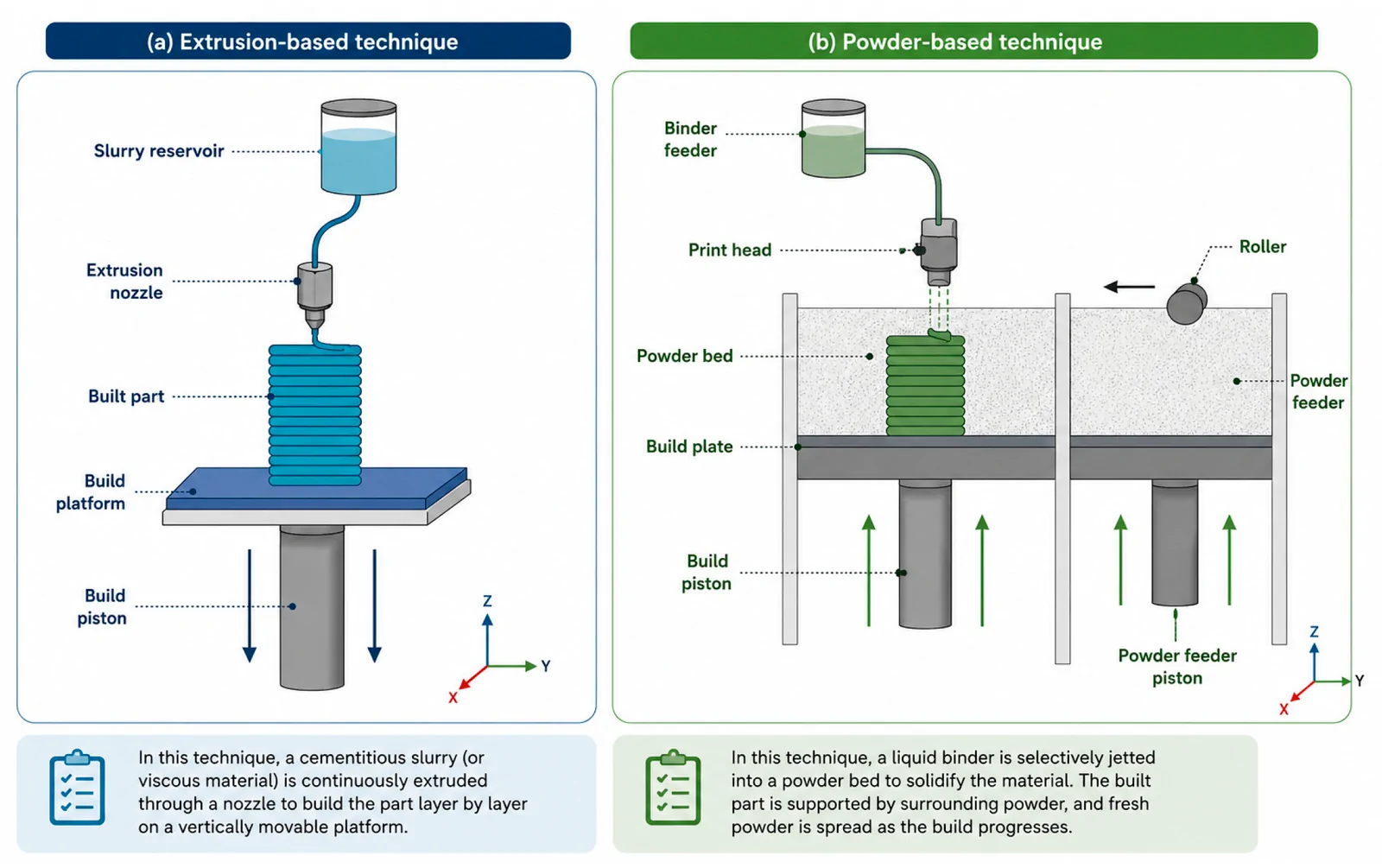
Printing techniques: (**a**) extrusion- and (**b**) powder-based techniques.

**Figure 3:**
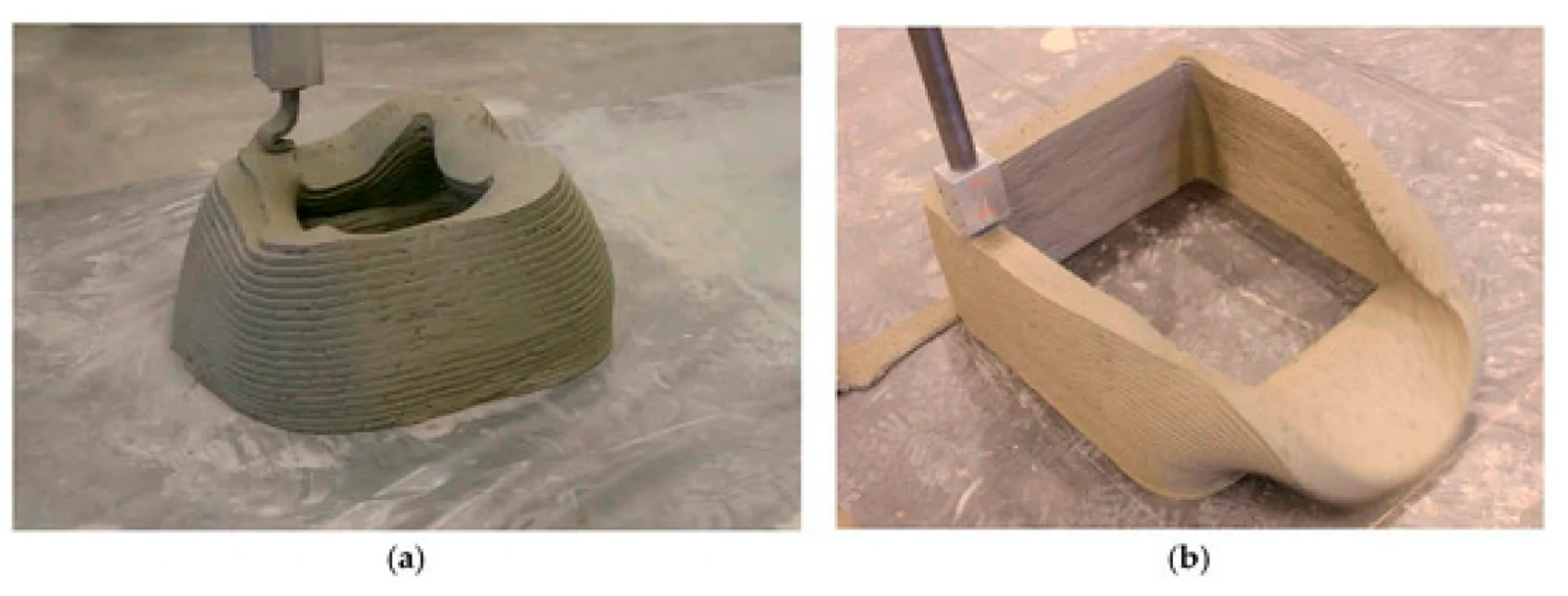
Principal structural failure modes during 3D concrete printing: (**a**) elastic buckling and (**b**) plastic collapse. Reproduced from Suiker et al. [59] under the CC BY 4.0 license.

**Figure 5:**
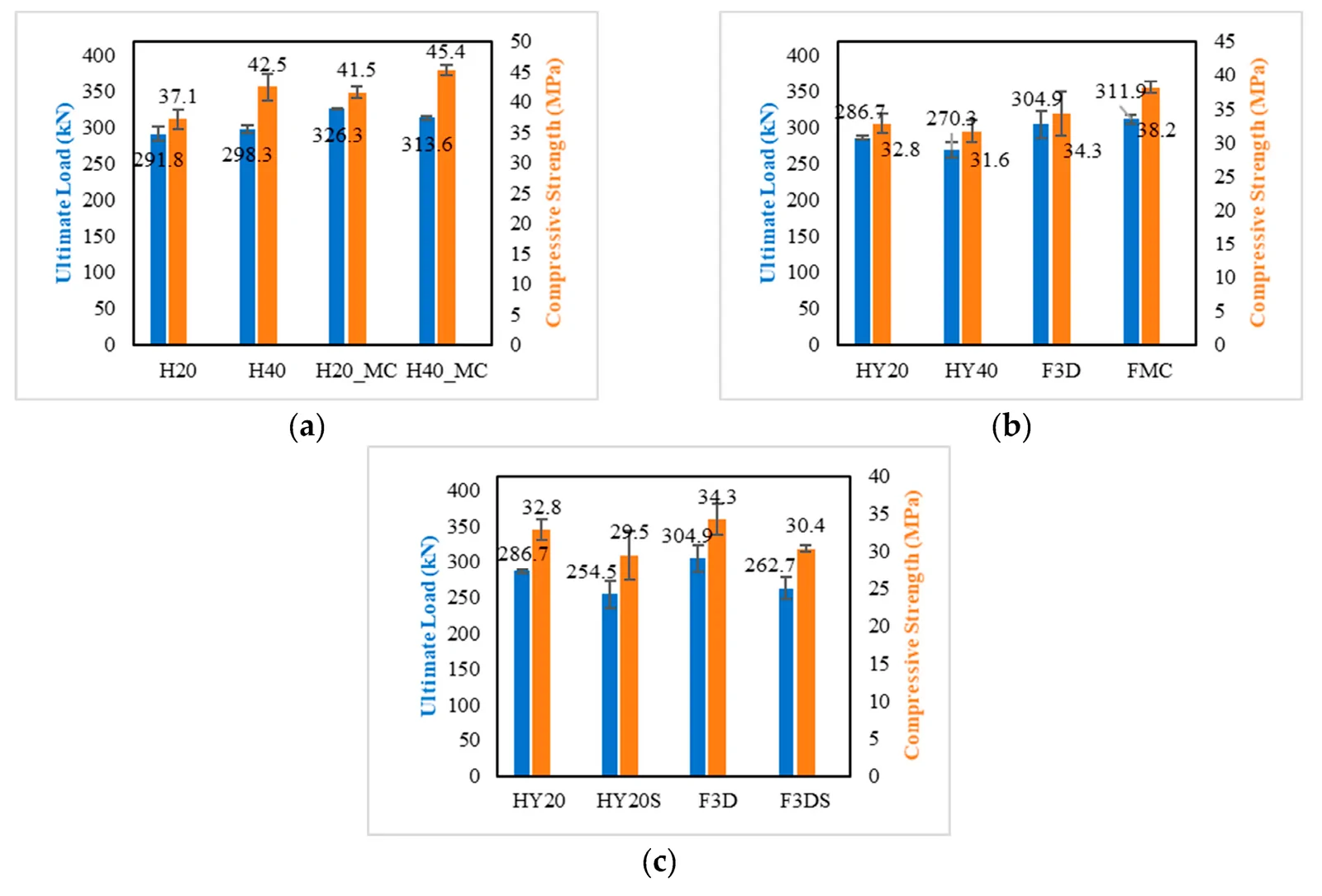
Axial compressive performance of 3D-printed circular elements with different configurations: (**a**) hollow printed-shell specimens with different void diameters, (**b**) solid specimens with hybrid, fully 3D-printed, and fully mold-cast configurations, and (**c**) solid specimens with staple reinforcement. Reproduced from Giwa et al. [71] under the CC BY 4.0 license.

**Figure 6:**
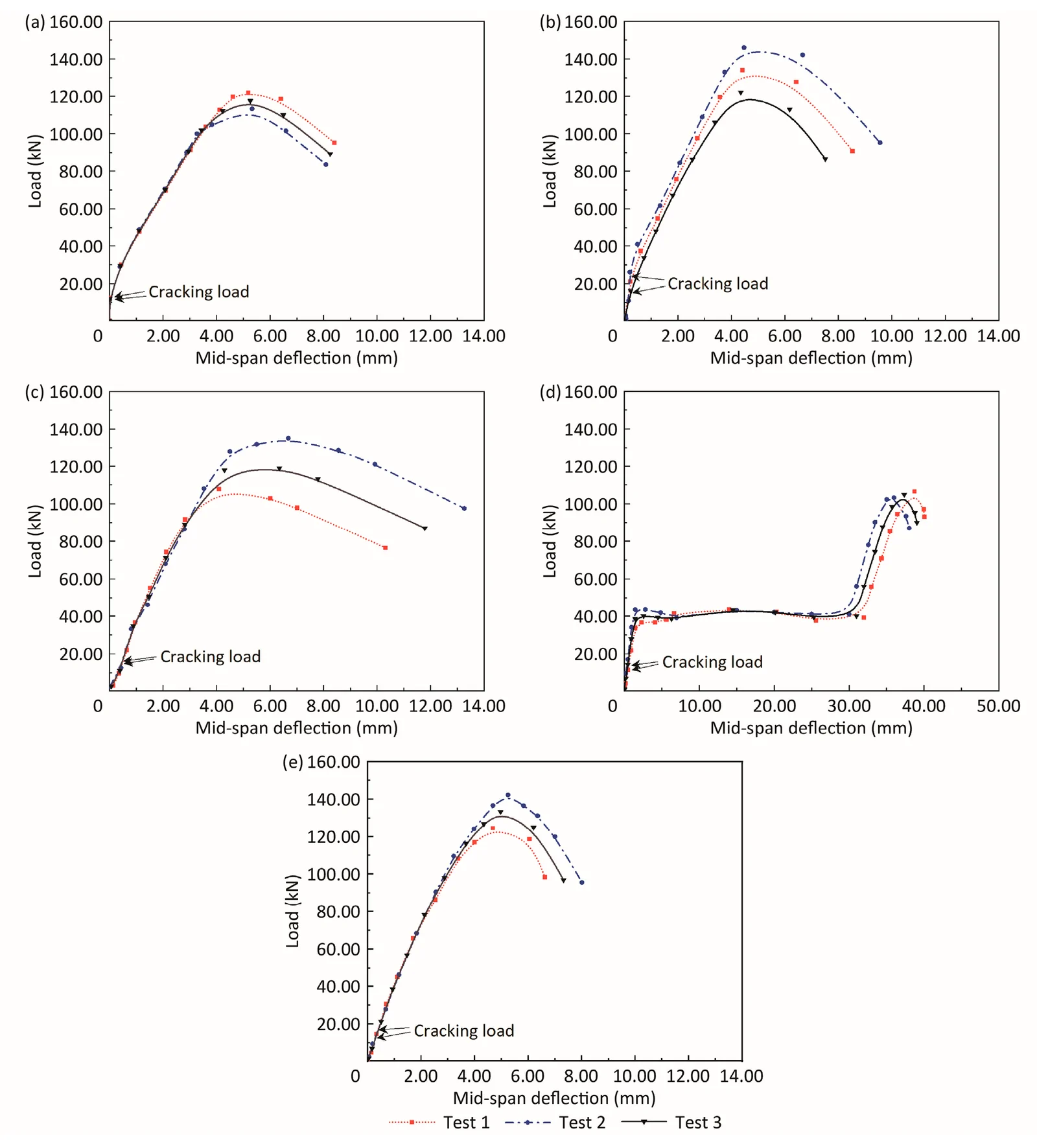
Load-deflection responses of reinforced concrete beams with different 3D-printed permanent formwork interface configurations: (**a**) MC, (**b**) NT, (**c**) SC, (**d**) MR, and (**e**) RB [63]. Reproduced from Guan et al. [63] under the CC BY 4.0 license.

**Figure 7:**
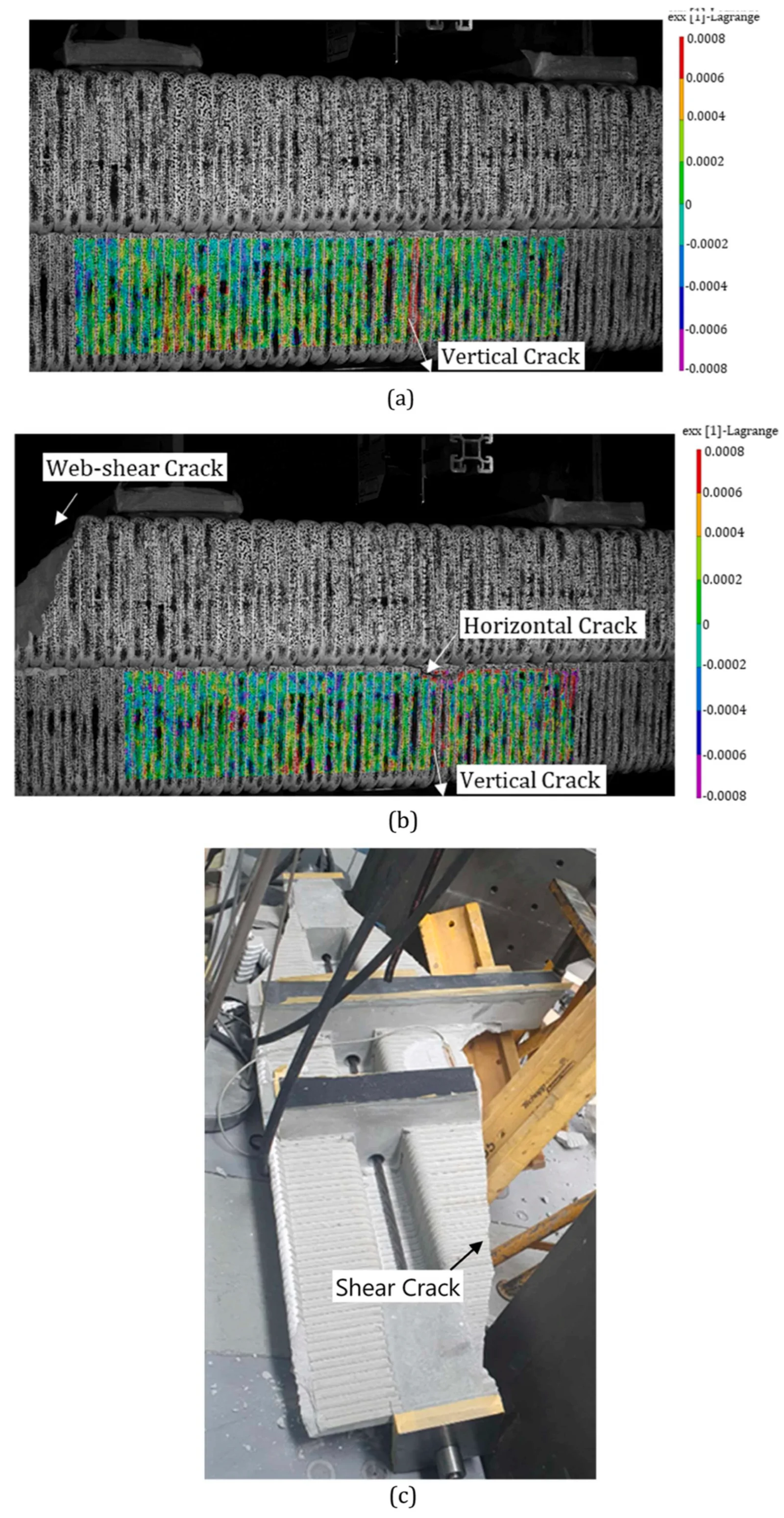
Crack initiation and damage progression of 3D-printed concrete stay-in-place formwork under four-point bending: (**a**) development of the first vertical flexural crack, (**b**) development of a web-shear crack, and (**c**) final failure associated with horizontal shear cracking [73]. Reproduced from Raza et al. [73] under the CC BY 4.0 license.

**Figure 8:**
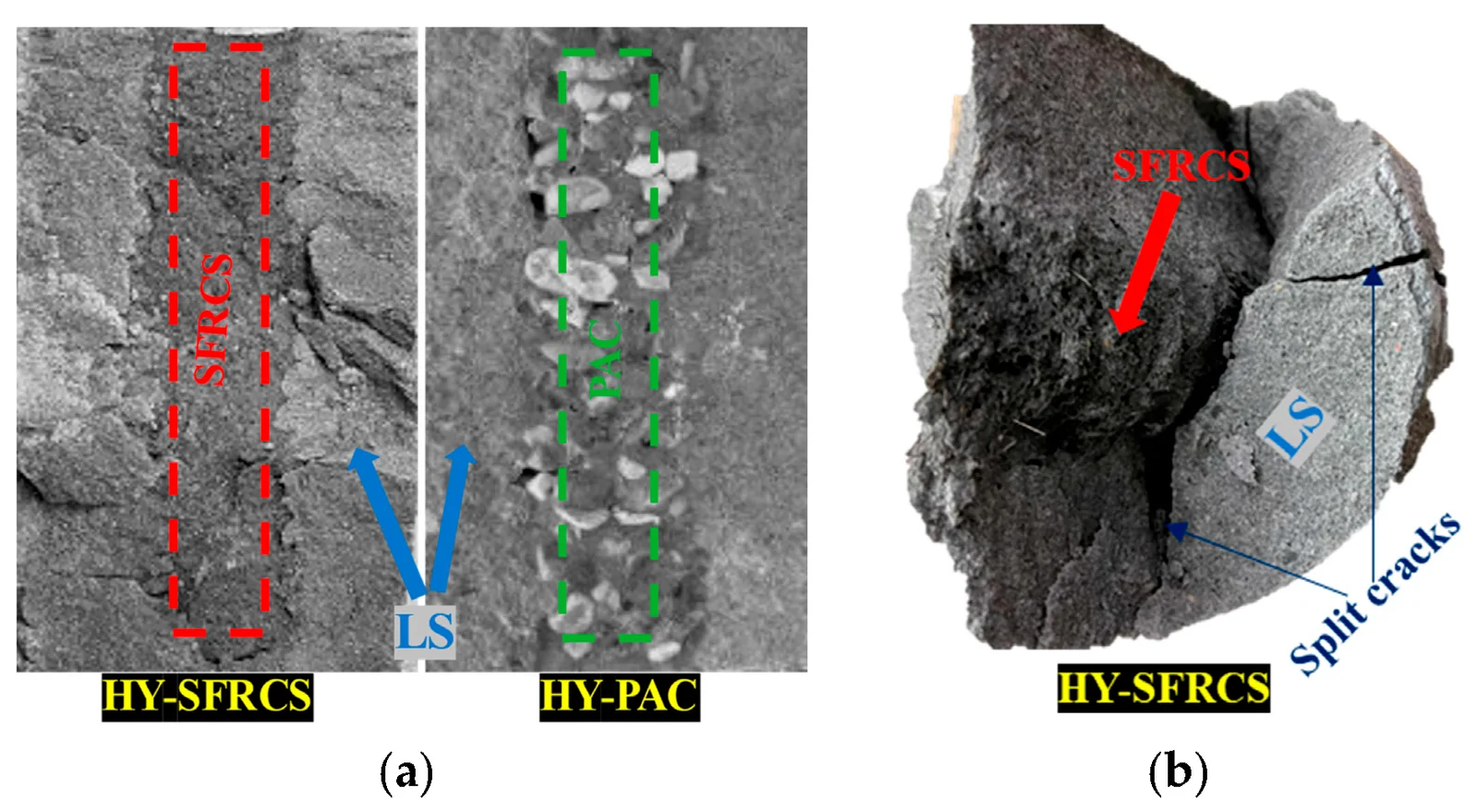
Interfacial characteristics of hybrid elements incorporating 3D-printed cementitious permanent formwork: (**a**) cross-section showing the interface between the 3D-printed shell and core material and (**b**) interfacial debonding observed after splitting tensile testing [71]. Adapted from Giwa et al. [71] under the CC BY 4.0 license.

**Figure 9:**
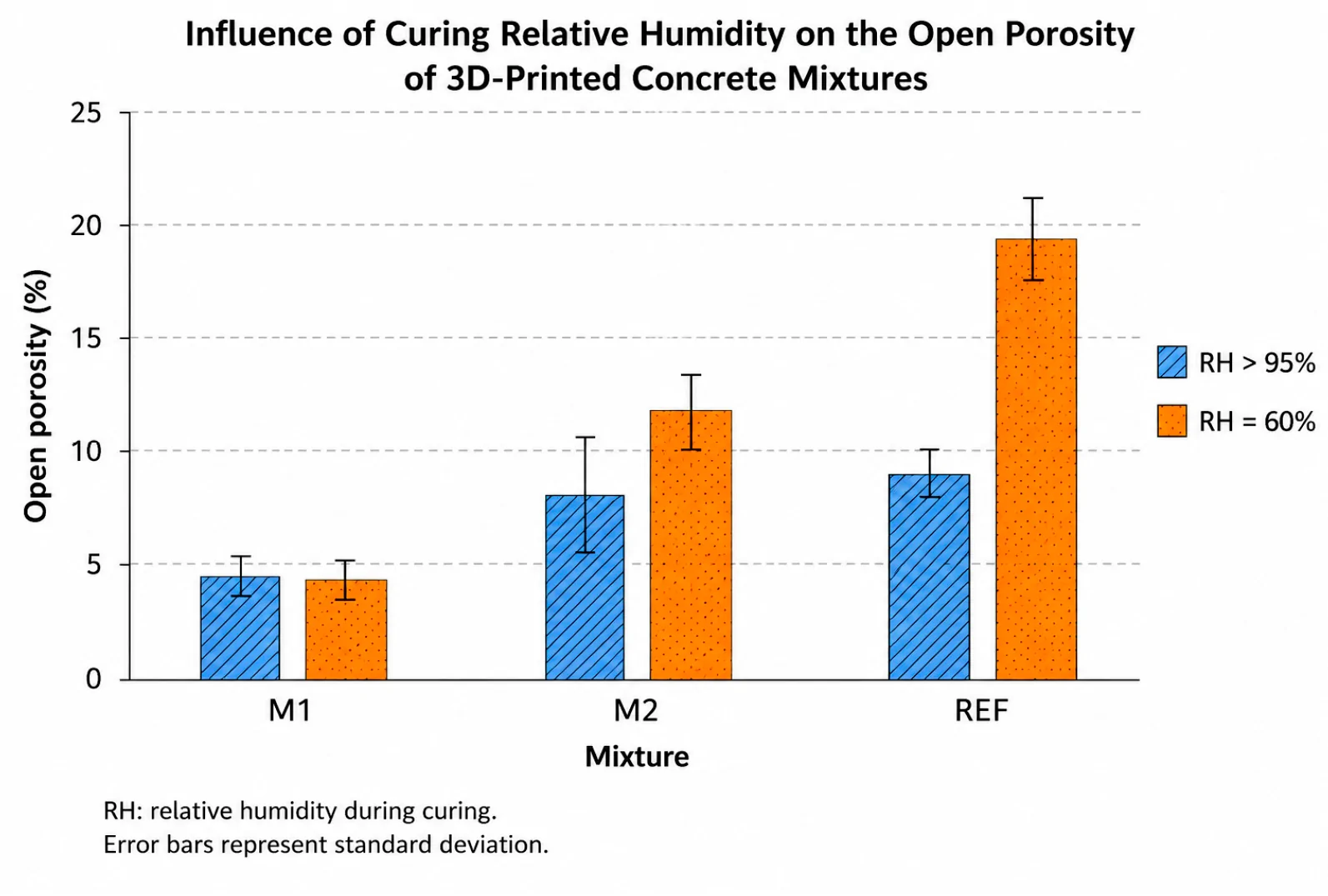
Open porosity of printed concrete under RH > 95% and RH = 60% for 28 days [65]. Adapted from Bekaert et al. [65] under the CC BY 4.0 license.

**Figure 10:**
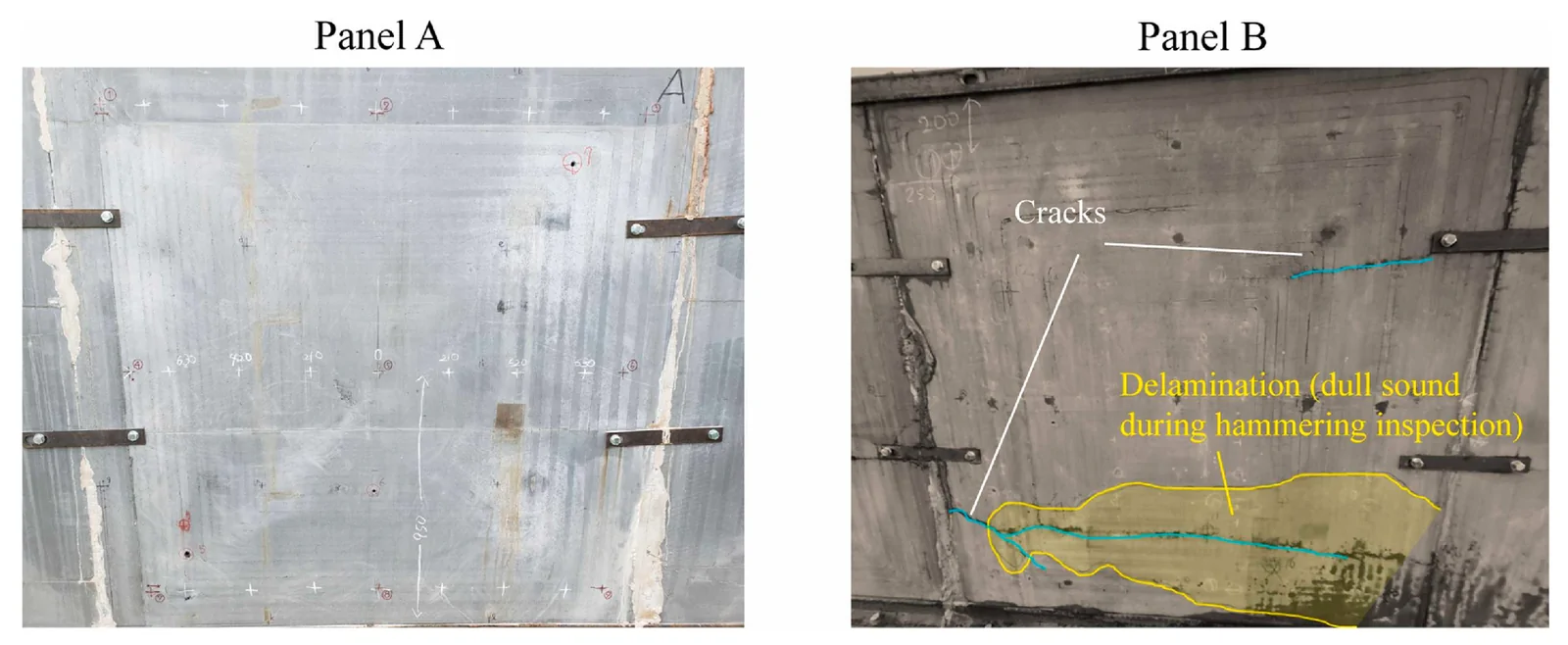
Visual and hammering inspection of 3D-printed permanent formwork panels. Panel A with no apparent surface defects and Panel B showing cracking and regions potentially affected by interlayer defects or delamination [79]. Reproduced from Shiotani et al. [79] under the CC BY 4.0 license.

**Figure 11:**
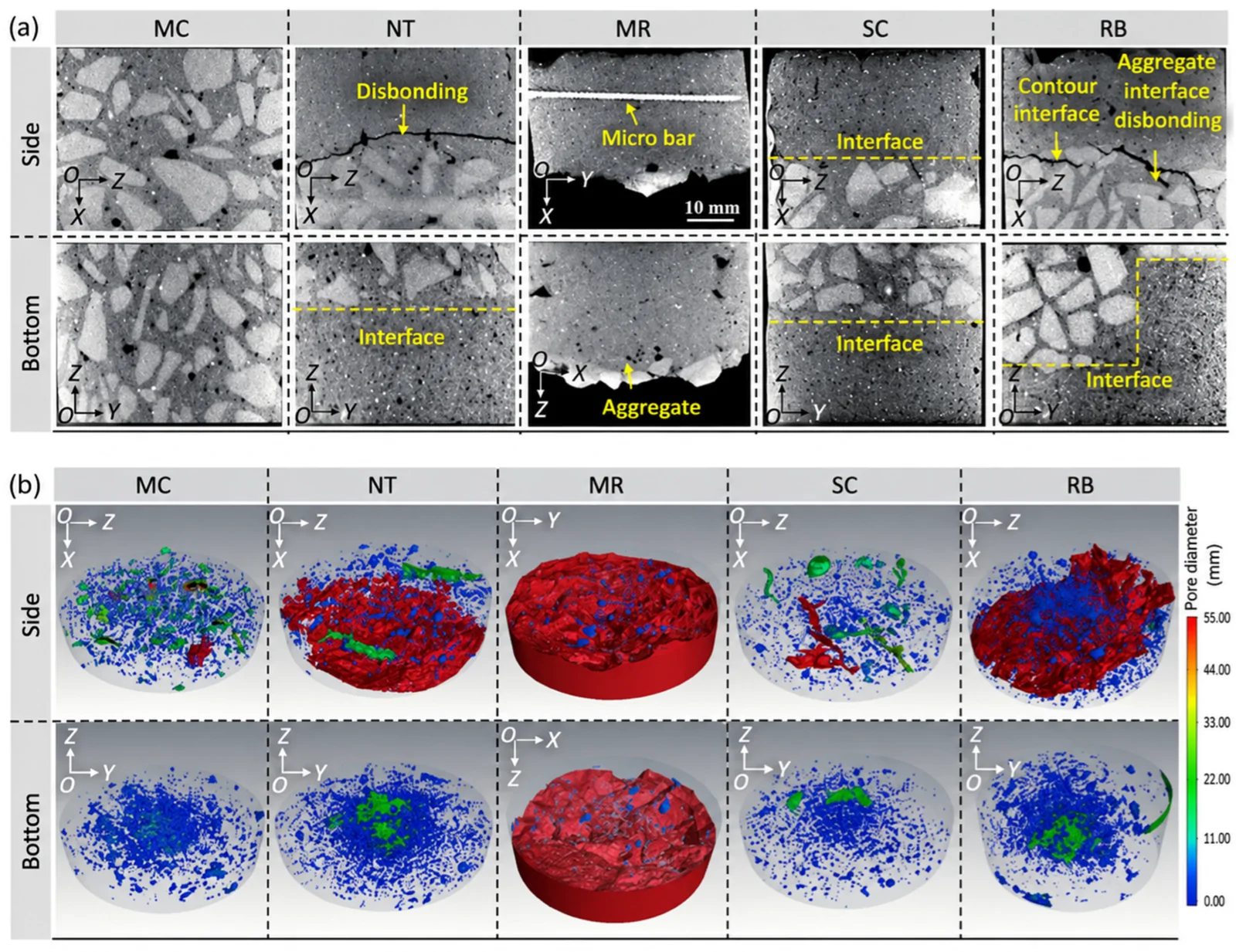
CT characterization of the interfaces between 3D-printed permanent formwork and cast- in-place concrete with different interfacial configurations [63]. (**a**) CT scan images; (**b**) 3D visualization of voids. Reproduced from Guan et al. [63] under the CC BY 4.0 license.

**Figure 12:**
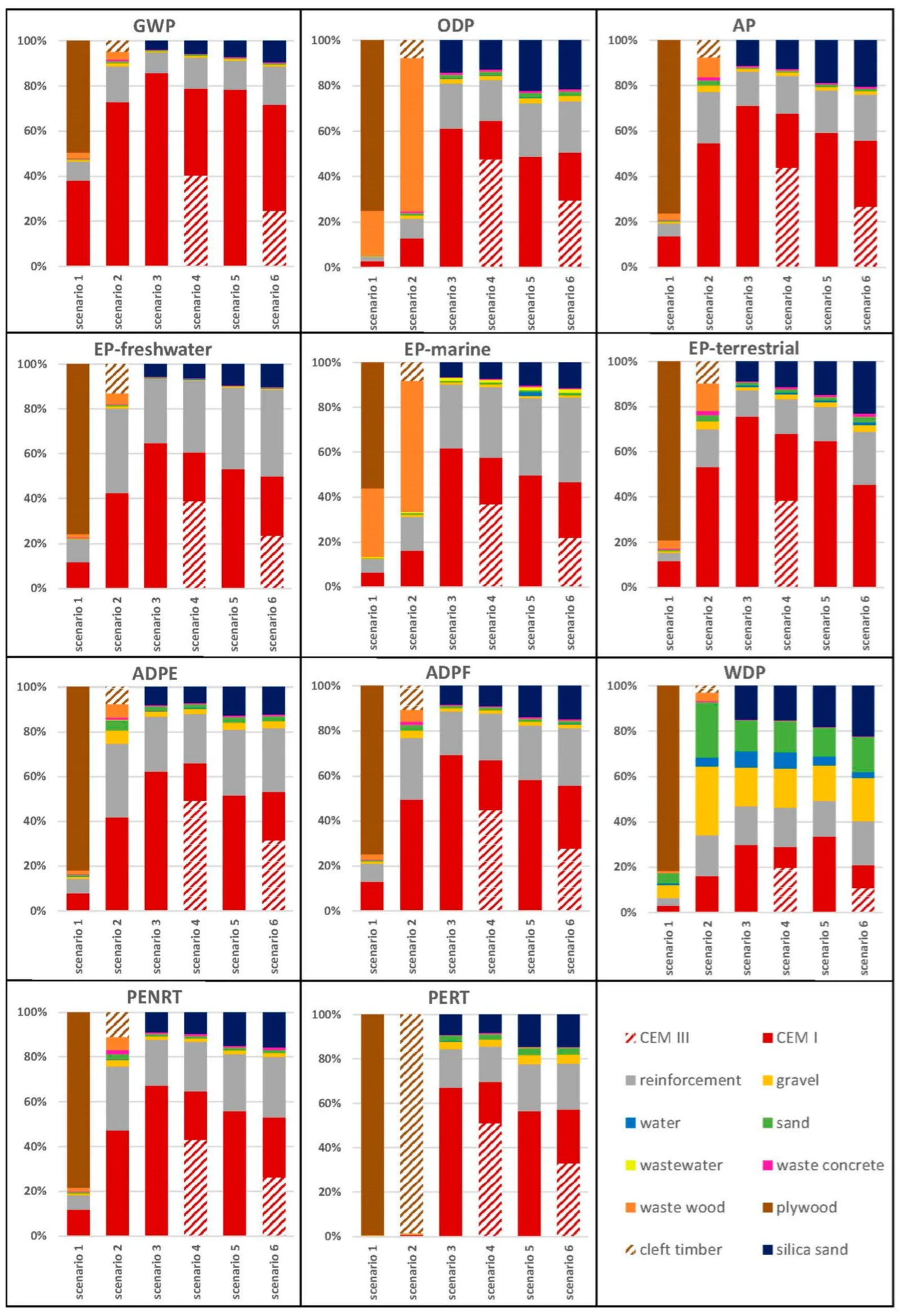
Material contributions to the environmental impacts of the six conventional and 3D-printed formwork scenarios [84]. Reproduced from Albrecht et al. [84] under the CC BY 4.0 license.

**Table 1:** Materials and structural applications investigated in 3DCP permanent formwork studies.

Study	3D-Printed Formwork Material (Binder/Aggregates/Fibers)	Element Printed or Tested
Zhu et al. [34]	Cementitious printable concrete; reinforcement ratios of 0%, 1.9%, and 2.5% were used in columns	Concrete columns; printed shell used as permanent formwork and filled with cast-in-place concrete. Good interface bonding and higher stiffness/bearing capacity than conventional columns were reported.
Wang et al. [38]	Cementitious 3D-printed formwork; variables included printed layer roughness of 10, 15, and 20 mm and age difference of 1, 3, 7, and 14 days	Interface bond specimens between 3D-printed formwork and post-cast concrete; tensile and shear bonding were evaluated.
Chen et al. [39]	Concrete with coarse aggregate; post-cast concrete with different coarse aggregate particle-size ratios	Interface bonding between 3D-printed concrete formwork and post-cast concrete.
Liu et al. [40]	PVA fiber-reinforced 3D concrete printing material; BFRP bars used as reinforcement	Composite columns reinforced with BFRP bars under compression.
Zhang et al. [41]	Cementitious 3D-printed concrete permanent formwork; unbonded post-tensioning used	Precast segmental concrete columns; cyclic behavior was studied.
Guan et al. [42]	3D-printed concrete permanent formwork with prestress reinforcement	Composite slabs; design, manufacturing, and structural performance were investigated.
Bai et al. [35]	ECC-based cementitious formwork; post-cast recycled concrete core	Composite beams; flexural behavior of 3D-printed ECC formwork with post-cast core recycled concrete.
Yamamoto et al. [43]	Short-fiber-reinforced 3D-printed concrete formwork	RC columns; seismic performance of columns surrounded by 3D-printed concrete permanent formwork.
Singh et al. [36]	SHCC cementitious shell; ductile fiber-reinforced material	Permanent formwork at prestressed girder ends to protect against cracking and environmental exposure.

Abbreviations: 3DCP, three-dimensional concrete printing; ECC, engineered cementitious composite; SHCC, strain-hardening cementitious composite; PVA, polyvinyl alcohol; BFRP, basalt fiber-reinforced polymer; RC, reinforced concrete.

**Table 4:** Mechanical characterization of 3DCP permanent formwork systems.

Study	Structural Level	Printed Shell	Specimen/Dimensions	Mechanical Characterization	Key Findings
Zhu et al. [34]	Composite column	3D-printed concrete permanent formwork	Columns: Ø250 × 500 mm; prisms: 40 × 40 × 160 mm; cylinders: Ø70 × 140 mm	Axial compression; load capacity, confinement, interface integrity, and failure mode	Printed shell enhanced confinement and axial load capacity; concave–convex interface improved load transfer and prevented interface peeling.
Liu et al. [40]	Composite column	PVA fiber-reinforced concrete permanent formwork	Columns: Ø250 × 500 mm	Axial and eccentric compression; composite action and interlayer cracking	Composite columns exhibited higher load-bearing capacity than conventional columns, although interlayer cracking developed under eccentric loading.
Bong et al. [45]	Composite beam	3DP-EGC permanent formwork	RC beams: 200 × 300 × 1800 mm	Four-point bending; flexural performance, ductility, crack distribution, and energy absorption	Strain-hardening geopolymer shell enhanced flexural performance, ductility, crack control, and energy absorption.
Guan et al. [63]	Composite beam	3D-printed concrete permanent formwork	Reinforced concrete beam specimens	Four-point bending; flexural performance and interface-assisted shear transfer	Anchor-interface geometry improved shear capacity by 81.4% while maintaining dimensional deviations within 5% of the design geometry.
Yu et al. [67]	Shell–core interface	3D-printed concrete permanent formwork	Splitting tensile: 100 × 100 × 100 mm; shear: 100 × 100 × 400 mm	Splitting tensile and four-point bending shear tests; interfacial bond	Physical grooving produced the highest bond performance through enhanced mechanical interlocking.
Wang et al. [38]	Shell–core interface	Sulfoaluminate cement permanent formwork	200 × 100 × 100 mm	Splitting tensile and antisymmetric four-point bending shear tests; interfacial bond	A 20 mm layer height and 7-day casting interval produced the highest interfacial bond performance.
Wang et al. [68]	Shell–core interface	3D-printed concrete permanent formwork	Cylinders: Ø74 × 36 mm	Splitting tensile and antisymmetric four-point bending shear tests; influence of casting concrete on bond	Self-compacting concrete with an expansion agent produced the strongest interface; epoxy coating reduced splitting tensile strength by 65.1%.
Bai et al. [69]	Shell–core interface	3D-printed ECC permanent formwork	200 × 100 × 100 mm	Splitting tensile and antisymmetric four-point bending shear tests; effects of printing parameters and material composition	A 15 mm layer height produced the optimum bond; 100% recycled sand reduced splitting tensile and shear strengths by 36.1% and 35.8%, respectively.
Qiu et al. [70]	Shell–core interface	3D-printed UHP-SHCC permanent formwork	Splitting tensile specimens	Splitting tensile test; interfacial tensile bond	Grooved and as-printed interfaces increased bond strength by 92.2–114.8% compared with a smooth interface.
Chen et al. [39]	Construction feasibility	Built-in-stirrup permanent formwork using printable coarse-aggregate concrete	Reinforced column formwork specimens	Structural evaluation; construction feasibility and applicability	Demonstrated the feasibility of coarse-aggregate 3D-printed permanent formwork with integrated stirrups for reinforced concrete columns.

Abbreviations: PVA, polyvinyl alcohol; RC, reinforced concrete; 3DP-EGC, 3D-printed engineered strain-hardening geopolymer composite; ECC, engineered cementitious composite; UHP-SHCC, ultra-high-performance strain-hardening cementitious composite.

**Table 5:** Comparison of interfacial bonding in 3DCP permanent formwork systems.

Study	Interface/Main Variable	Casting Interval	Test	Main Bond Result
Wang et al. [38]	Layer height: 10, 15, 20 mm	1, 3, 7, 14 days	Tensile; shear	20 mm and 7 days provided the best bond performance
Yu et al. [67]	Untreated, cement paste, grooved, combined	1, 3 days	Splitting tensile; shear	Grooving improved bond; 1 day generally >3 days
Wang et al. [68]	Post-cast concrete type; epoxy treatment	Not reported	Splitting tensile; shear	Expansion-agent SCC gave strongest interface; epoxy reduced tensile bond
Bai et al. [69]	Layer height, fiber content, recycled sand	Not reported	Splitting tensile; shear	15 mm optimum; 100% recycled sand reduced bond by 36.1%/35.8%
Qiu et al. [70]	Smooth, grooved, as-printed	Not reported	Splitting tensile	Grooved/as-printed increased bond by 92.2–114.8%
Chen et al. [74]	Post-cast aggregate grading	Not reported	Splitting tensile	Smaller aggregate improved interface filling and bond

## Data Availability

No new data were created or analyzed in this study. Data sharing is not applicable to this article.
